# An integrative approach to the anatomy of *Syllis gracilis* Grube, 1840 (Annelida) using micro-computed X-ray tomography

**DOI:** 10.7717/peerj.7251

**Published:** 2019-07-08

**Authors:** Julio Parapar, Carlos Caramelo, María Candás, Xela Cunha-Veira, Juan Moreira

**Affiliations:** 1 Departamento de Bioloxía, Universidade da Coruña, A Coruña, Spain; 2 Estación de Bioloxía Mariña da Graña, Universidade de Santiago de Compostela, Ferrol, Spain; 3 Departamento de Biología, Universidad Autónoma de Madrid, Madrid, Spain

**Keywords:** Annelida, Syllidae, Micro-CT, Histological sectioning, Scanning electron microscopy, Anatomy

## Abstract

**Background:**

The overall anatomy of the genus *Syllis* (Annelida: Syllidae) has been largely studied; however, an integrative approach considering different anatomical techniques has never been considered. Here, we use micro-computed X-ray tomography (micro-CT) to examine the internal anatomy of *Syllis gracilis* Grube, 1840, along with other widely available techniques.

**Methods:**

We studied the anatomy of the marine annelid *S. gracilis* through an integrative approach, including micro-CT along with stereo and light compound microscopy (STM, LCM), scanning electron microscopy (SEM), confocal laser scanning microscopy (CLSM) and histological sectioning (HIS). In this manner, we evaluated the applicability of micro-CT for the examination of annelid anatomy by testing whether the images obtained make it possible to visualize the main body structures, in comparison with other current techniques, of the various elements of its internal anatomy.

**Results:**

Overall external and internal body elements are clearly shown by the integrative use of all techniques, thus overcoming the limitations of each when studied separately.****Any given method shows disparate results, depending on the body part considered. For instance, micro-CT provided good images of the external anatomy, including relevant characters such as the shape, length and number of articles of dorsal parapodial cirri. However, it is especially useful for the examination of internal anatomy, thus allowing for 3D visualization of the natural spatial arrangement of the different organs. The features best visualized are those of higher tissue density (i.e., body musculature, anterior parts of the digestive tract), particularly in 3D images of unstained specimens, whereas less electrodense tissues (i.e., the peritoneal lining of septa and nervous system) are less clearly visualized. The use of iodine stain with micro-CT has shown advantages against non-staining for the adequate observation of delicate elements of low density, such as the segmental organs, the connective between the ganglia, the ventral nerve cord and segmental nerves.

**Discussion:**

Main external anatomical elements of *S. gracilis* are well shown with micro-CT, but images show lesser optical resolution and contrast when compared to micrographs provided by SEM and CLSM, especially for fine structural features of chaetae. Comparison of micro-CT and HIS images revealed the utility and reliability of the former to show the presence, shape and spatial disposition of most internal body organs; the resolution of micro-CT images at a cellular level is, however, much lower than that of HIS, which makes both techniques complementary.

## Introduction

Syllidae Grube, 1850 is one of the most speciose and morphologically diverse families of Annelida, currently with 74 genera and nearly 700 valid species described (San Martín & Aguado, 2014). Most of the previous taxonomic and phylogenetic studies have been based on the examination of external anatomy characters by means of stereomicroscopy, light microscopy and scanning electron microscopy (e.g., Aguado, Nygren & Siddall, 2007; Aguado & San Martín, 2009; Nygren, 1999; San Martín, López & Aguado, 2009; Álvarez Campos et al., 2015; San Martín & Worsfold, 2015).

*Syllis gracilis* (Grube, 1840) is a well-known syllid species, originally described in the Gulf of Naples, and commonly reported in a number of substrates in shallow temperate coastal waters around the world (Imajima, 2003; Gil, 2011). It is mostly characterised by the presence of Y-shaped chaetae (i.e., ypsiloid chaetae) that result from the fusion of the shaft and blade of falciger compound chaetae (San Martín, 2003). This feature is, however, shared with other taxa, that have been considered as junior synonyms of this species, regardless of the large geographic distances between them (see Licher, 1999); this resulted in considering *S. gracilis* as cosmopolitan, although this has been questioned by recent molecular phylogenetic studies (e.g., Maltagliati et al., 2000; Álvarez Campos, Giribet & Riesgo, 2017).

In comparison to other annelids, syllid species identification requires no detailed anatomical studies (i.e., dissection and/or histological sectioning; Fernández et al., 2014). Because of their small size, the study of internal syllid anatomy relies on the identification of larger elements, such as the anterior part of the digestive tract—e.g., the eversible axial-type pharynx, *aka* proboscis, and the conspicuous highly muscularised proventricle - which are often visible through the body wall or by histological sectioning (San Martín, 2003; San Martín & Aguado, 2014). Therefore, the study of most of the internal characters was traditionally limited to purely anatomical works rather than to systematic and phylogenetic ones. Previous work on syllid anatomy has included *in vivo* observations under the stereomicroscope (STM), light compound microscopy (LCM), histological sectioning (HIS) techniques and scanning electron microscopy (SEM) (see San Martín & Aguado, 2012). More recently, cytochemical approaches with Confocal Laser Scanning Microscopy (CLSM) and Transmission Electron Microscopy (TEM) have improved our knowledge of their internal anatomy (e.g., Helm & Capa, 2015; Weidhase et al., 2016; Weidhase et al., 2017).

The micro-computed X-ray tomography (micro-CT) has been demonstrated to be a powerful, fast, complementary and novel tool for exploring internal anatomy across many metazoan taxa, including terrestrial and marine vertebrates and invertebrates (Metscher & Müller, 2011; Handschuh et al., 2013; Fernández et al., 2014; Sombke et al., 2015). For instance, its usefulness in studying Annelida anatomy has recently been tested (see Dinley et al., 2010; Faulwetter et al., 2013a; Faulwetter et al., 2013b; Paterson et al., 2014; Parapar et al., 2017a) and increasingly used in descriptions of new taxa (e.g., Parapar, Moreira & Helgason, 2015; Parapar et al., 2016; Parapar et al., 2017b). However, none of these works compares their results with those obtained with other techniques, such as the examination of histological sections (but, see Parapar et al., 2018). This would be necessary to truly assess the strengths and limitations of this novel technique when used in taxonomy and systematics. On the other hand, micro-CT *per se* is a non-invasive technique and, therefore, seems suitable for studying the anatomy of preserved material (including type specimens) stored in museums and other collections, thus avoiding any significant alteration.

We studied the external and internal anatomy of *Syllis gracilis* using different available anatomical methods and tested the capabilities of micro-CT in the study of general annelid anatomy. To this end, several specimens collected from the same location in NW Spain were analysed with micro-CT to cover the potential individual variability of the taxon, along with the integrative use of different macroscopic, microscopic and staining methods, including STM and LCM, SEM, CLSM and HIS, the latter by using two staining procedures. Transmission Electron Microscopy (TEM) and CLSM at histological and cellular level were not explored here (see ‘Discussion’). Therefore, by considering this multi-specimen integrative approach, cross validating of techniques could be achieved, and misinterpretations of any given structure (i.e., observational artefacts) could also be potentially avoided. Images obtained through these techniques were compared with those of micro-CT in terms of quality, contrast and reliability, and assessing whether these techniques are complementary or otherwise. Furthermore, specimens selected were previously treated and fixed under standard conditions, as in many benthic studies (i.e., with ethanol 70% or formalin 4%), not following any special protocol. This will make it possible to test if micro-CT provides acceptable results with material not specifically prepared for this technique, as occurs with most specimens stored in museums and research institutions.

## Materials & Methods

Specimens of *Syllis gracilis* were collected in the Ria of Ferrol, Galicia (NW Atlantic coast of the Iberian Peninsula) in the framework of several benthic studies (Table S1). This species is the most abundant syllid at this location from the intertidal to 25 m depth (Parapar et al., 1996). A total of 18 specimens were examined and about half of them were selected from a collection obtained between 2000 and 2002 from several intertidal rocky shore sites. Additional specimens were collected in 2008, 2012 and 2015 in shallow subtidal sediments in Santa Lucía bay, located in the southern margin of the ria. Samples were taken by (1) scraping the macroalgae on the rocky surface and (2) by means of a Van-Veen grab in sedimentary bottoms. Samples were sieved through a 1-mm mesh, specimens sorted to species level, fixed in 10% formalin and finally transferred to 70% ethanol for preservation.

Additional material was collected between 2000 and 2002 and had been previously deposited in the Museo Nacional de Ciencias Naturales-MNCN (Madrid) by the first author with the registration numbers MNCN16.01/15999-16001, MNCN16.01/16003, MNCN16.01/16007 and MNCN16.01/16011-16013. Several specimens used for description under stereomicroscope and light microscope, HIS, SEM, CLSM and micro-CT were extracted from this collection and have received new registration numbers: MNCN 16.01/18341 to MNCN 16.01/18409 and MNCN 16.01/18412 to MNCN 16.01/18415 (Table S1). Two more specimens, later collected in Santa Lucía Bay (see above), were used for micro-CT and deposited in the MNCN with reg. numbers MNCN 16.01/18410 and MNCN 16.01/18411. Sampling coordinates and abiotic features of all samples are given in Table S1. Preparatory treatment data for histological sections, and micro-CT and scanning parameters used for each specimen studied under this technique and CLSM are shown in Tables S2 to S4.

Identification of specimens, drawings and measurements were performed using an Olympus SZX9 stereomicroscope and an Olympus BX40 compound microscope, both equipped with camera lucida at the Universidade da Coruña (UDC), Universidad Autónoma de Madrid (UAM) and Estación de Bioloxía Mariña da Graña, Universidade de Santiago de Compostela (EBMG, USC). Photographs were taken with a compound light microscope Nikon Eclipse 90i equipped with Nomarsky Differential Interference Contrast (DIC).

Examination with the SEM and CLSM was performed at the Servizos de Apoio á Investigación-SAIN (UDC) (*Research Support Services*). For SEM, specimens were dehydrated via a graded ethanol series; critical-point dried using CO_2_, covered with gold in a BALTEC SCD 004 evaporator, and examined and photographed under a JEOL JSM-6400 SEM (see Parapar et al., 2017a). For CLSM, four specimens previously fixed in formalin and stored in 70% ethanol were washed with 0.1 M phosphate buffered saline (PBS, pH 7.4). After 3 rinses for 15 min, specimens were permeabilized with 0.5% Triton X-100 in 0.1 M PBS for 10–15 min. To stain muscular tissue, specimens were incubated in a solution containing phalloidin-FITC (5–10 µl stock solution in 500 µl PBS; Sigma-Aldrich) during 90–120 min at room temperature. Subsequently, samples were washed in PBS for 5 min and mounted between two coverslips with a drop of glycerol/PBS, 1:1. Specimens were analysed with a confocal laser microscope Nikon A1; image stacks were processed with Nis-Elements (Nikon). Parameters used with each specimen are indicated in Table S2.

Histological sectioning and staining were performed at UDC and EBMG, respectively. To this end, specimens previously preserved in 70% ethanol were dehydrated through a series of graded ethanol baths and clearing in xylene, infiltrated with paraffin at 57 °C overnight, and embedded in a paraffin block. Blocks were sectioned with a microtome in 8–10 µm sections, which were placed on microscope slides, hydrated and stained with Hematoxylin-Eosin or Mallory trichromic, dehydrated, and finally mounted on permanent slides with Canada balsam. For Hematoxylin-Eosin stained sections, slides were submerged in Harris hematoxylin for 15 min, washed in tap water for 10 min and counterstained with eosin for 2 min. For Mallory stained sections, slides were submerged in acid fuchsine for 15 min and air dried for 25 min. Once dry, they were submerged in aniline blue-orange G solution for 20 min and washed briefly in baths of water and ethanol. Parameters used with each specimen are indicated in Table S3.

Examination with micro-CT was conducted at the EBMG. Specimens were gradually dehydrated in ascending ethanol series up to 96% ethanol. Selected specimens were stained with 1% iodine in 96% ethanol for three days. All specimens were dehydrated for two hours with hexamethyldisilazane (HMDS) and left to dry overnight before scanning, following Alba-Tercedor & Sánchez-Tocino (2011). Scanning was conducted with a microtomograph Skyscan 1172. Parameters used with each specimen are indicated in Table S4. Samples were rotated 360° to obtain as much detail as possible. The X-ray projection images obtained during scanning were reconstructed with the NRecon software (Bruker, Kontich, Belgium). The sections obtained were processed with CTAn and DataViewer software (Bruker, Kontich, Belgium) and 3D representations with CTVox (Bruker, Kontich, Belgium) software. To present the results in a homogeneous manner—and thus facilitate comparison between species and with results of previous works, 2D sections and 3D renderings were shown as coronal (frontal), sagittal and transversal views, both from stained or non-stained specimens, following Parapar et al. (2017a). Images were edited with Corel Draw software in order to enhance the contrast. Datasets of transverse 2D images of studied specimens were uploaded at the Morphosource repository (https://www.morphosource.org/Search/Index?search=Syllis).

## Results

### External morphology and species delimitation

**Figure 1:**
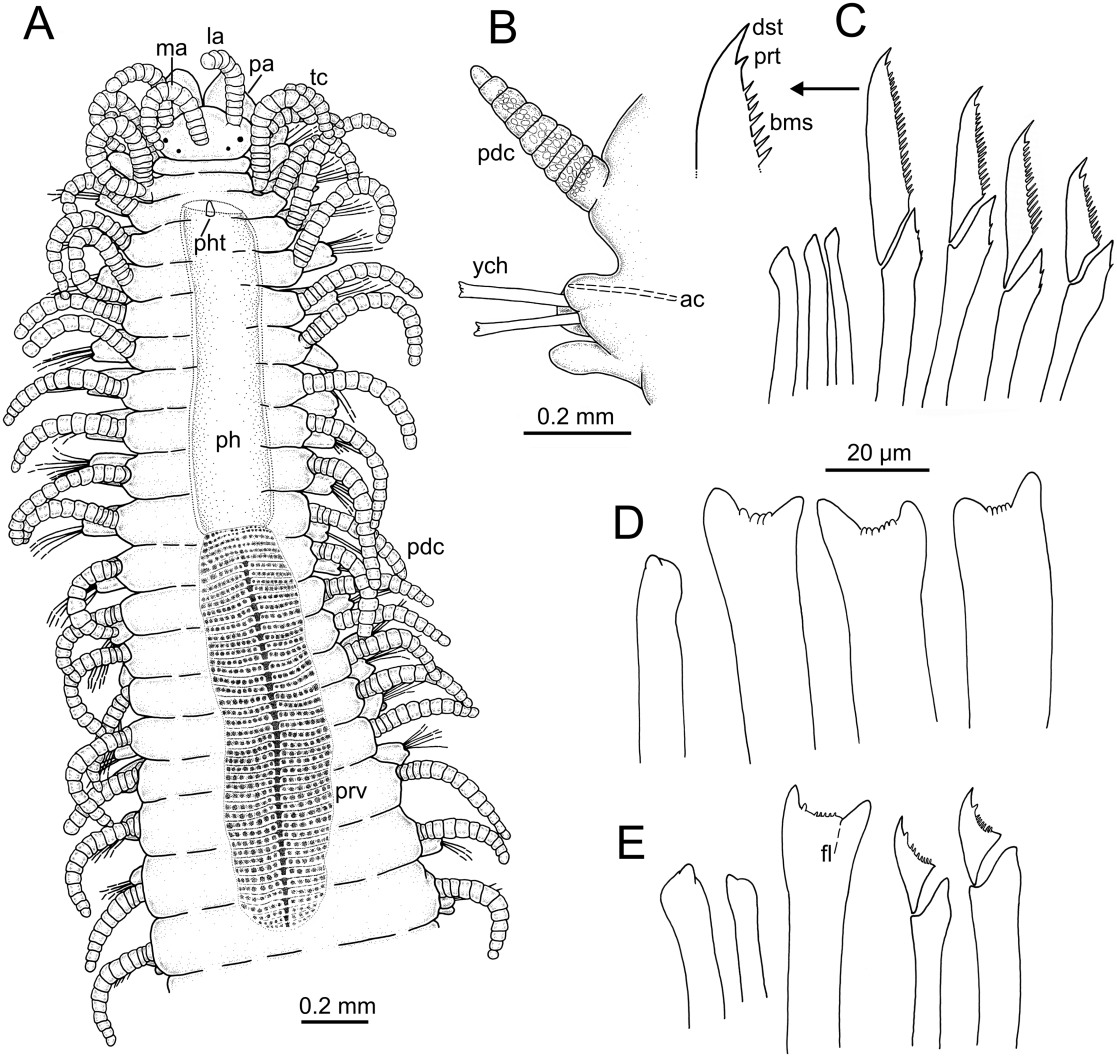
Line drawings of *Syllis gracilis*. One specimen showing main characters used in traditional taxonomic descriptions (MNCN 16.01/18405). (A) Anterior end, dorsal view; (B) mid-body parapodium; (C) aciculae (left) and compound falcigers (right) of third parapodium; detail: blade distal end; (D) acicula (left) and ypsiloid chaetae (right) of a mid-body parapodium; (E) aciculae (left), ypsiloid chaeta (middle) and compound falcigers (right) of a mid-posterior parapodium.

**Figure 2:**
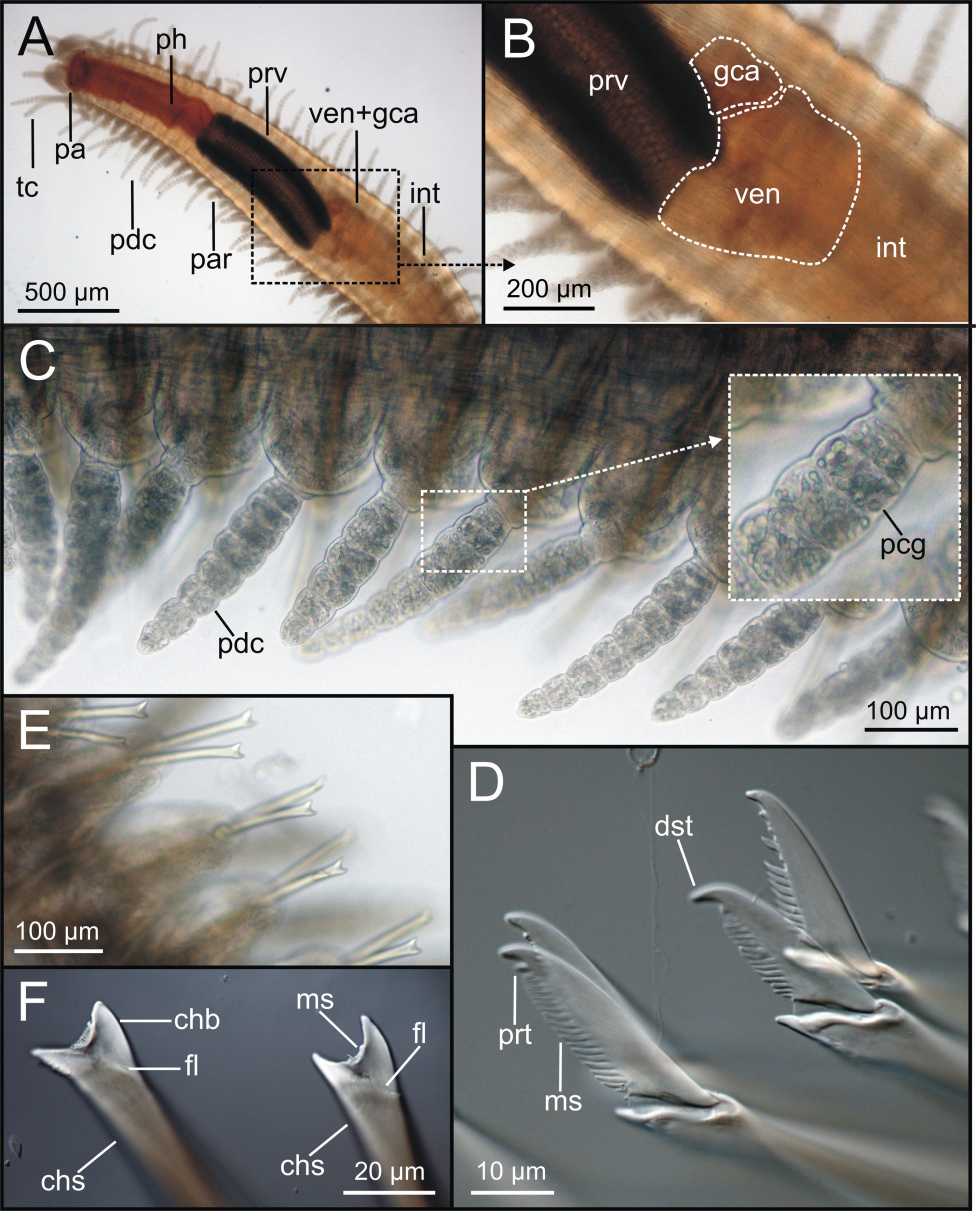
LCM micrographs of *Syllis gracilis*. Images at low-medium-high magnification of one specimen (MNCN 16.01/18368-18372). (A) Anterior end, dorsal view (40 ×) showing main gut regions as seen by transparency; (B) proventricle-ventricle-intestine region; ventricle and gastric caeca areas highlighted by broken lines (200 ×); (C) detail of mid-body parapodial cirri (200 ×); insert showing several cirrus articles with glands inside; (D) anterior parapodium, compound chaetae (400 ×); (E) mid-body parapodia, ypsiloid chaetae (200 ×); (F) detail of two mid-body ypsiloid chaetae showing main chaetal elements (1000 ×).

**Figure 3:**
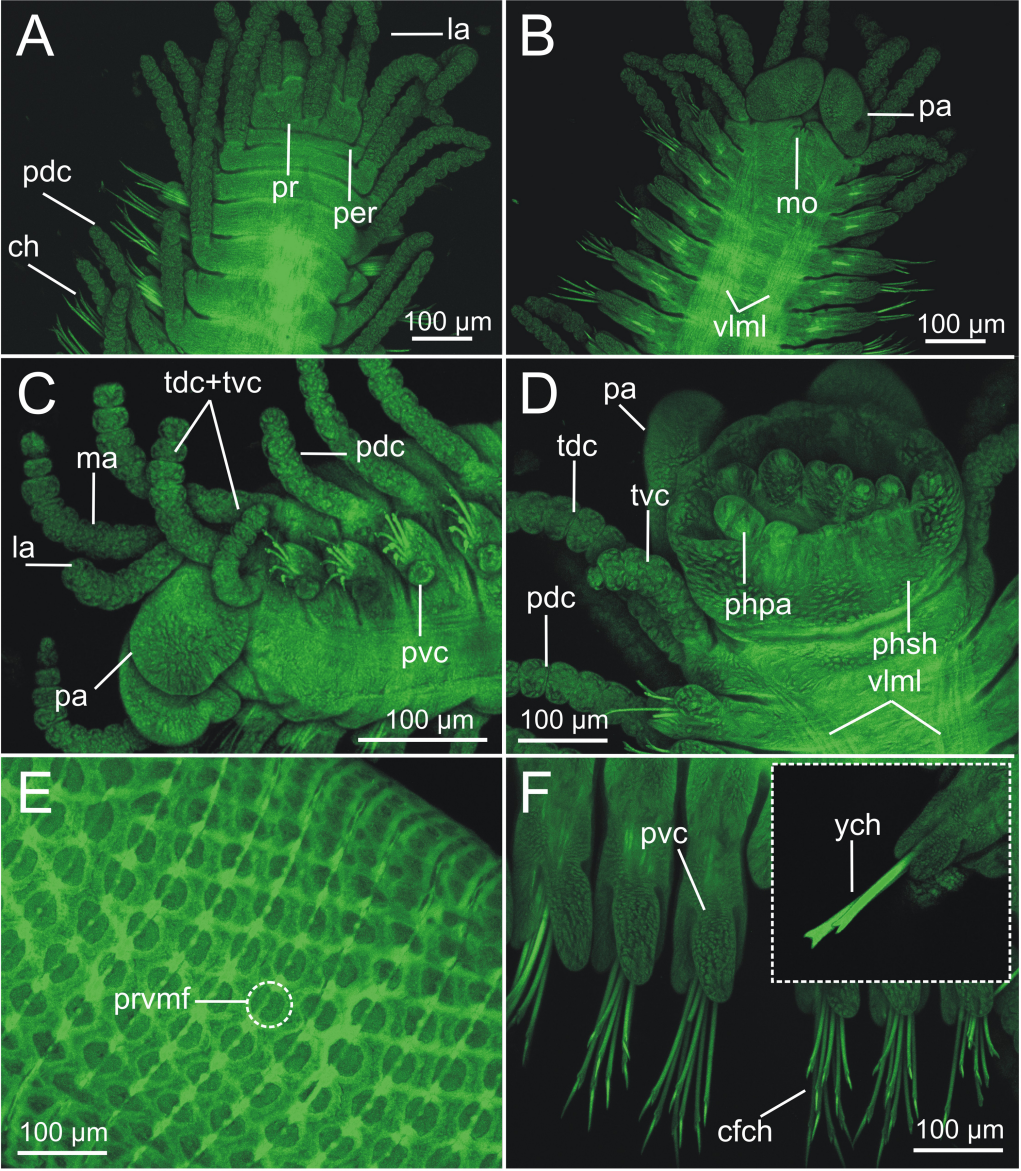
CLSM micrographs of *Syllis gracilis*. Two specimens, showing mainly relevant morphological external elements (MNCN 16.01/18410, MNCN 16.01/18414). (A) Anterior end, dorsal view (100 ×); (B) anterior end, ventral view (100 ×); (C) anterior end, left ventro-lateral view (100 ×); (D) anterior end, ventral view, showing anterior end of pharynx projecting from buccal cavity (200 ×); (E) right anterior chaetigers in ventral view (200 ×); insert showing two ypsiloid chaetae; (F) proventricle extracted from body showing surface main elements (200 ×).

**Figure 4:**
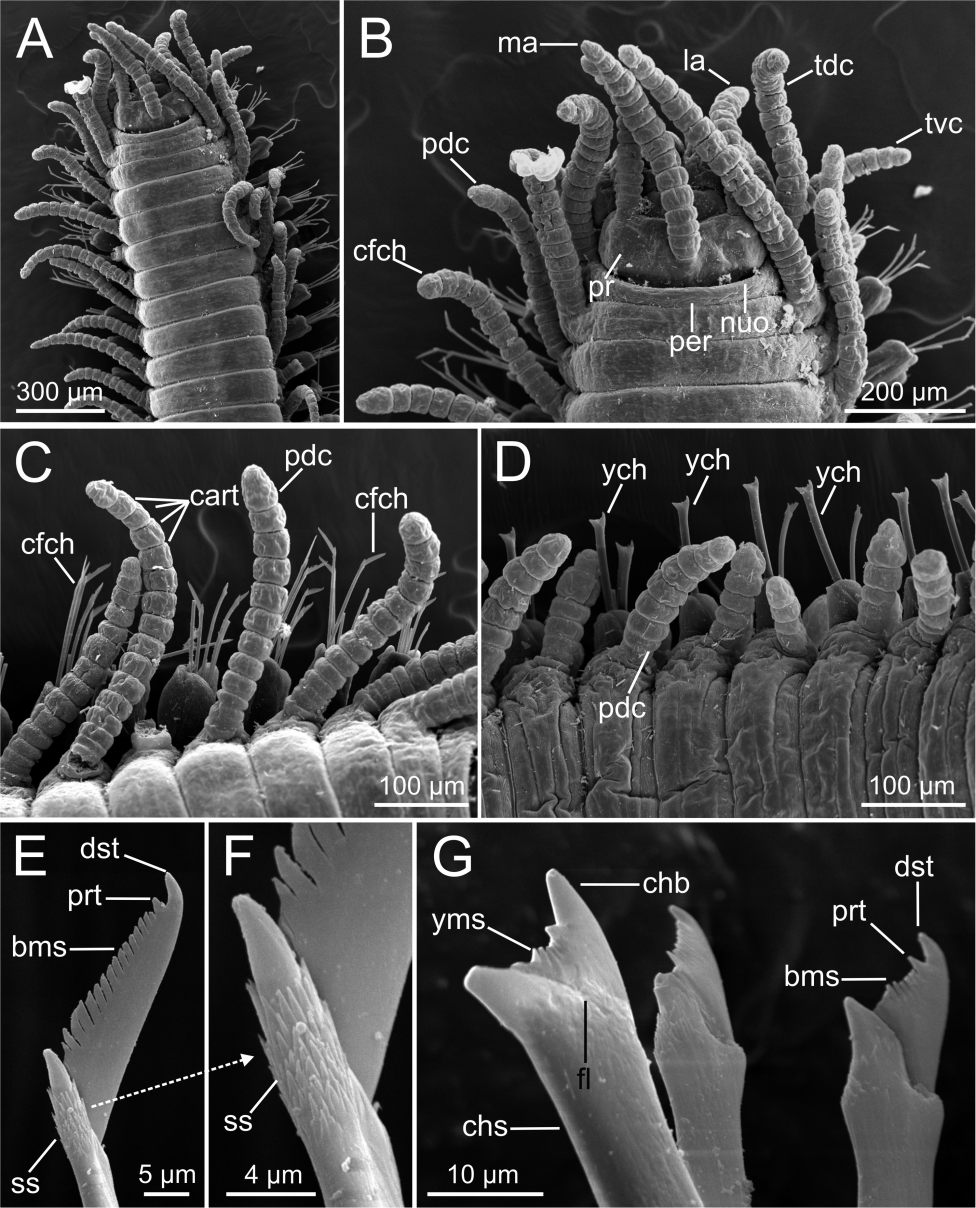
SEM micrographs of *Syllis gracilis*. One specimen (MNCN 16.01/18341). (A) Anterior end, dorsal view; (B) detail of anterior end showing main sensory elements; (C–D) detail of anterior and median region in dorsal view showing several parapodial cirri and chaetae; (E) compound long-blade falciger chaeta; (F) detail of shaft distal end of falciger chaeta shown in E; (G) one ypsiloid and two compound short-blade chaetae from mid-anterior chaetiger.

**Figure 5:**
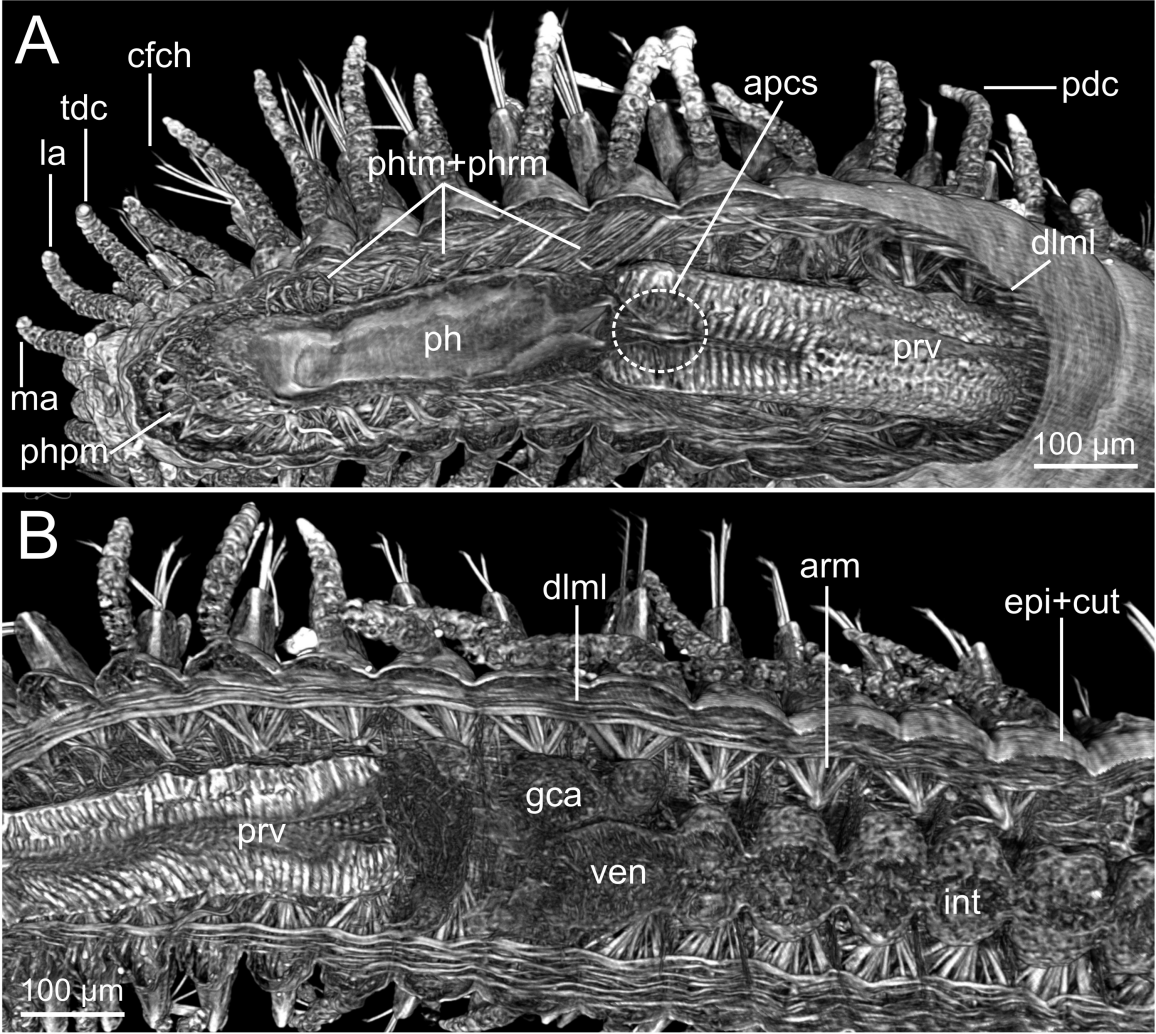
Micro-CT 3D volume renderings of *Syllis gracilis*. External and internal anatomy of an unstained specimen (MNCN 16.01/18342-18349). (A) Frontal section at pharynx-proventricle region showing musculature associated with protraction and retraction; (B) frontal section at proventricle-ventricle-intestine region showing main gut parts and musculature associated with parapodial movement.

**Figure 6:**
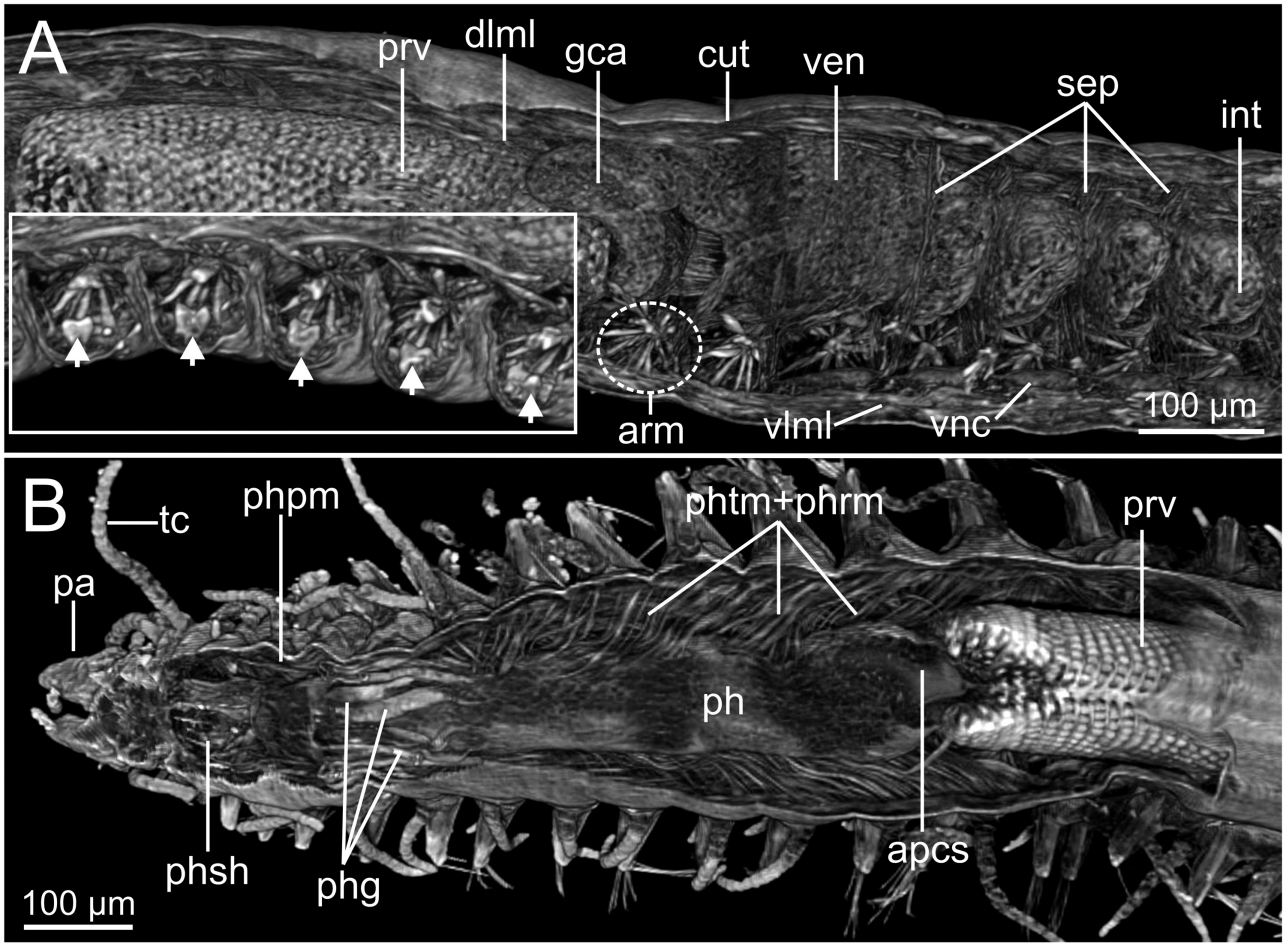
Micro-CT 3D volume renderings of *Syllis gracilis*. Internal anatomy of two unstained specimens (MNCN 16.01/18410 and MNCN 16.01/18411). (A) Composition of two pictures of sagittal section at proventricle-ventricle-intestine region; insert showing the position of segmental organs (arrowheads) below parapodial aciculae and acicular retractor muscles; (B) frontal section at pharynx-proventricle region showing pharyngeal retractor and protractor muscles.

**Figure 7:**
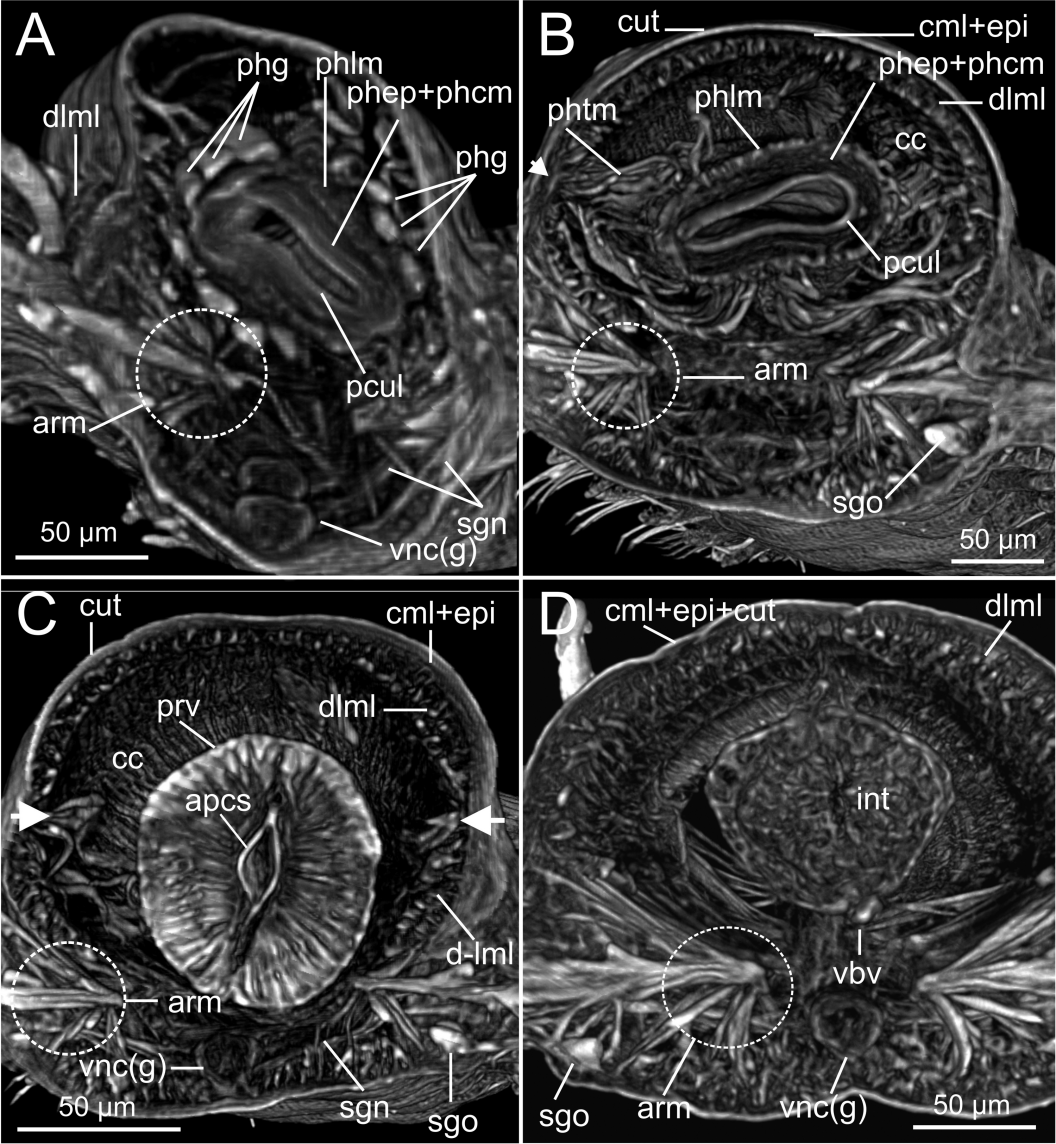
Micro-CT 3D volume renderings of *Syllis gracilis*. Internal anatomy of two unstained specimens of *Syllis gracilis* (MNCN 16.01/18410 and MNCN 16.01/18411). (A–D) Four transversal sections at anterior and posterior pharyngeal, proventricle and intestine regions respectively showing main body elements. Arrowheads in B and C showing point of attachment of pharyngeal retractor muscles to inner body wall.

Examination of external characteristics of specimens revealed that they mostly agree with previous descriptions of *Syllis gracilis* from the Iberian Peninsula (cfr. San Martín, 2003, pp. 413–416, fig 226, 227). Distinctive recognised characters were: (1) complete specimens ranged from 2 to 29 mm in length and with 15 to 156 chaetigers (Figs. 1A, 2A); (2) anterior segments of fixed specimens show light brown pigmentation arranged in two dorsal horizontal lines per segment; (3) pharynx similar in length to proventricle, extending through 9 segments, and proventricle bearing around 45 muscle fibres (Fig. 1A); (4) antennae, tentacular cirri and dorsal parapodial cirri about as long as body width, with 12–15 articles (Figs. 1A, 1B, 3A–3D, 4A–4C); (5) mid-body and posterior ones are much shorter and spindle-shaped, with 5–9 articles (Figs. 2B, 2C, 4D); (6) anterior parapodia with 4–5 bidentate falciger chaetae with a short secondary tooth (Figs. 1C, 2D, 4E, 4F); (7) mid-body chaetigers with 1–2 (up to five) ypsiloid chaetae each (Figs. 1B, 1D, 2E, 2F, 3E, 4G), with fusion line between blade and shaft sometimes distinguishable, and 0 (sometimes up to 2) compound falcigers; (8) posterior chaetigers with 1–3 (normally 2) ypsiloid chaetae and 0–3 falciger chaetae with short bidentate blades with a short secondary tooth (Fig. 1E); (9) three distally curved aciculae in each anterior parapodium (Fig. 1C), and two aciculae of similar shape from mid-body to posterior end (Figs. 1D, 1E).

Micro-CT images referred to external body features show a similar definition to those obtained with the stereomicroscope, but with less detail than those of both optic microscope (Fig. 2), CLSM (Fig. 3) and SEM (Fig. 4). Main external characters can be discernible with micro-CT (i.e., shape and body length, number and length of articles in antennae, tentacular and parapodial cirri) (Figs. 5A–5B, 6B), but with much less resolution, which is even poorer for other finer and highly relevant taxonomic characters such as ypsiloid and compound chaetae.

STM images also lack detail in finer body structures (i.e., chaetae) but, in turn, show relevant taxonomic information in characters such as the shape and length of parapodial dorsal cirri as well as dorsal body colouration and head features (prostomium, palps, eyes). But however, such images are always of a similar or lower quality than those provided by micro-CT.

LCM (Fig. 2), CLSM (Fig. 3) and SEM (Fig. 4) images show better detail than micro-CT and STM regarding chaetae and some features of internal anatomy that can be seen by transparency of the body wall. Low-medium magnification (40 × to 400 ×) in LCM shows general external anatomy (Fig. 2A), head, and shape and number of articles of dorsal parapodial cirri and whether there is a pattern of alternation in length of cirri (Fig. 2C). They also make it possible to observe several internal organs through the very thin and transparent integument, particularly the gut (Figs. 2A, 2B), the extension (i.e., number of chaetigers) of each region such as the pharynx and the proventricle (Fig. 2A), and the presence of small features (e.g., pharyngeal tooth), the proventricle muscular fibres, and the presence and size of ventricle and gastric caeca (ven, gca, Fig. 2B) that connect the proventricle to the intestine. Higher magnification (1,000×) allows for detailed examination of other highly relevant taxonomic finer features such as the presence and shape of the parapodial aciculae, marginal spines of distal blade of compound falcigers (Fig. 2D) and the ypsiloid chaetae (Figs. 2E, 2F). The latter cannot be observed in such detail by means of the micro-CT.

CLSM images (Fig. 3) show that, in general, external characters are better defined than with LCM. On the contrary, SEM images show more resolution than CLSM. Furthermore, laser scanning and subsequent layer integration result in 3D images while providing high superficial definition (e.g., Figs. 3A–3D). This is not true for chaetae when compared with LCM at high magnification (cfr. Figs. 3E vs. 2E, 2F). Examination of internal structures extracted through dissection benefits from this technique; for example, this makes it possible to distinguish the organization of the muscular fibres in the proventricle (Fig. 3F).

SEM allows for the most accurate examination of external body features by providing the highest detail of body surface (Figs. 4A–4D) while revealing taxonomic characters hardly visible with other techniques, such as the presence of nuchal organs that appear as short ciliated grooves behind the prostomium (Fig. 4B). SEM images show the chaetal architecture with maximum detail in comparison to other techniques. Some chaetal features revealed in this manner are the shape and length of blade teeth in the compound chaetae, and the presence of spines in their shaft (Fig. 4E, 4F) and a fusion line between shaft and blade in the ypsiloid chaetae (Fig. 4G).

### Internal anatomy

The combined approach of two complementary imaging methods such as micro-CT and HIS (Figs. 5–15) provided an integrative view of the internal anatomy of the studied specimens.

### Integument and musculature

**Figure 8:**
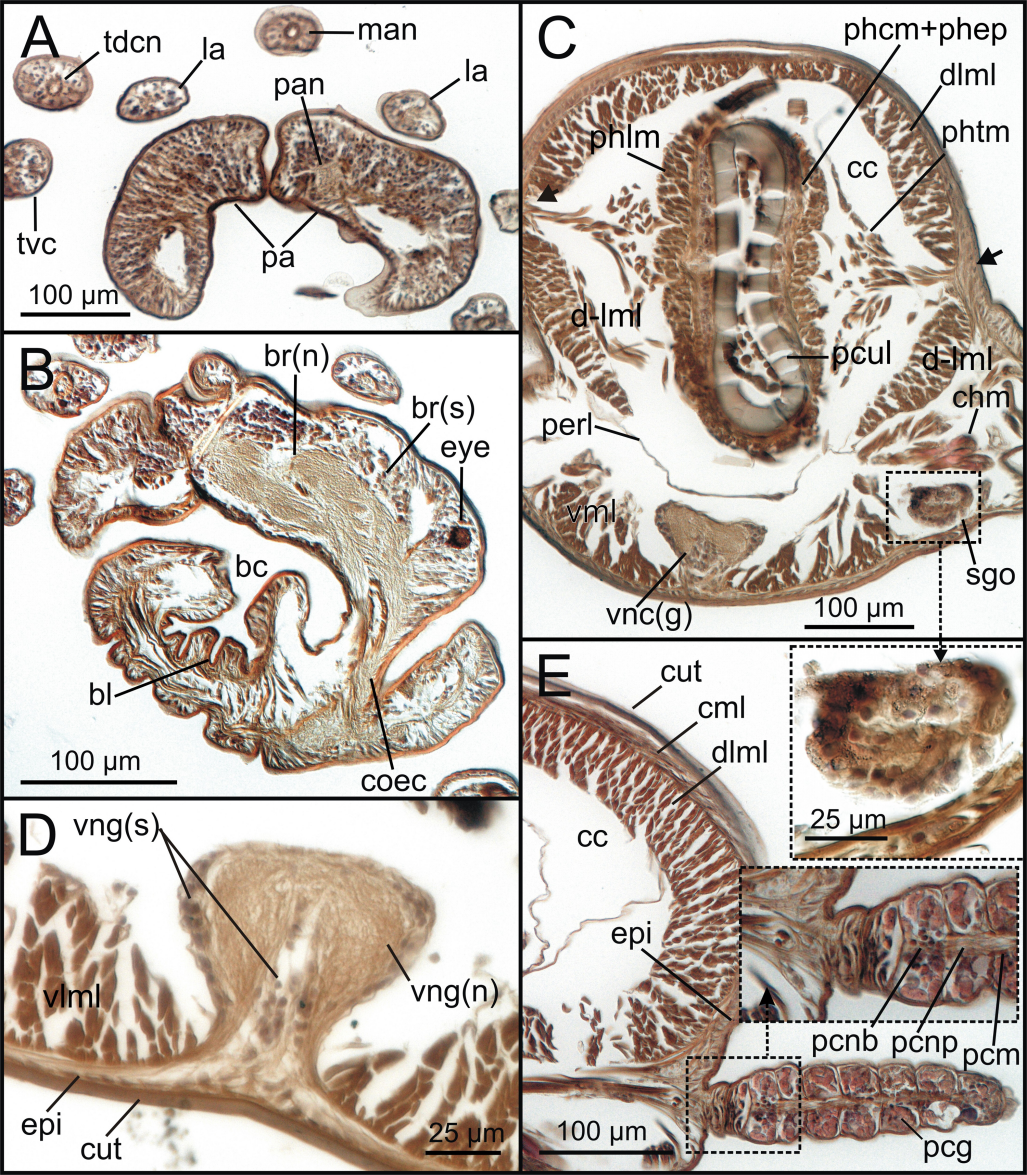
Transversal histological sections of *Syllisgracilis*. Two specimens stained with Hematoxylin-Eosin (MNCN 16.01/18342-18349 and MNCN 16.01/18350-18367). (A) Palps and several sensory head elements; (B) prostomium and buccal cavity showing brain, left eye and left circumoesophageal connective; (C) pharynx, showing pharyngeal retractor muscles, ventral nerve cord ganglia and left segmental organ; framed: segmental organ; (D) detail of a ventral nerve cord ganglion showing differentiation between soma and axonic parts of neurons; (E) left median hemi-metamere at intestine level showing continuous dorsal muscle layer and muscular and nervous elements associated with dorsal parapodial cirrus; framed: basal part of parapodial cirrus.

The cuticle is clearly seen in coronal (frontal) (cut, Figs. 5B, 6B), para-sagittal (Fig. 6A) and transversal micro-CT images (Figs. 7A–7D). On the contrary, the epidermis (with the basal membrane) and the circular muscle layer (epi, cml) are poorly portrayed, and seen as an apparent “empty space” between the cuticle and the underlying longitudinal muscular layer (dlml, Fig. 7B), but not in the iodine stained specimen where a continuum is observed in its place (Fig. 15C). HIS, however, shows a far better definition of such layers (Fig. 8C, 8E, 11A–11B), especially at higher magnifications (Fig. 8D). The arrangement of the integument components is visible in the longitudinal sections of the beaded parapodial dorsal cirrus, including the secretory cell glands, muscular fibres, nerve bodies and processes (pcg, pcm, pcnb, pcnp, Fig. 8E); micro-CT images do not, however, clearly show the glandular elements inside parapodial dorsal cirri (pdc, Figs. 5A–5B).

**Figure 9:**
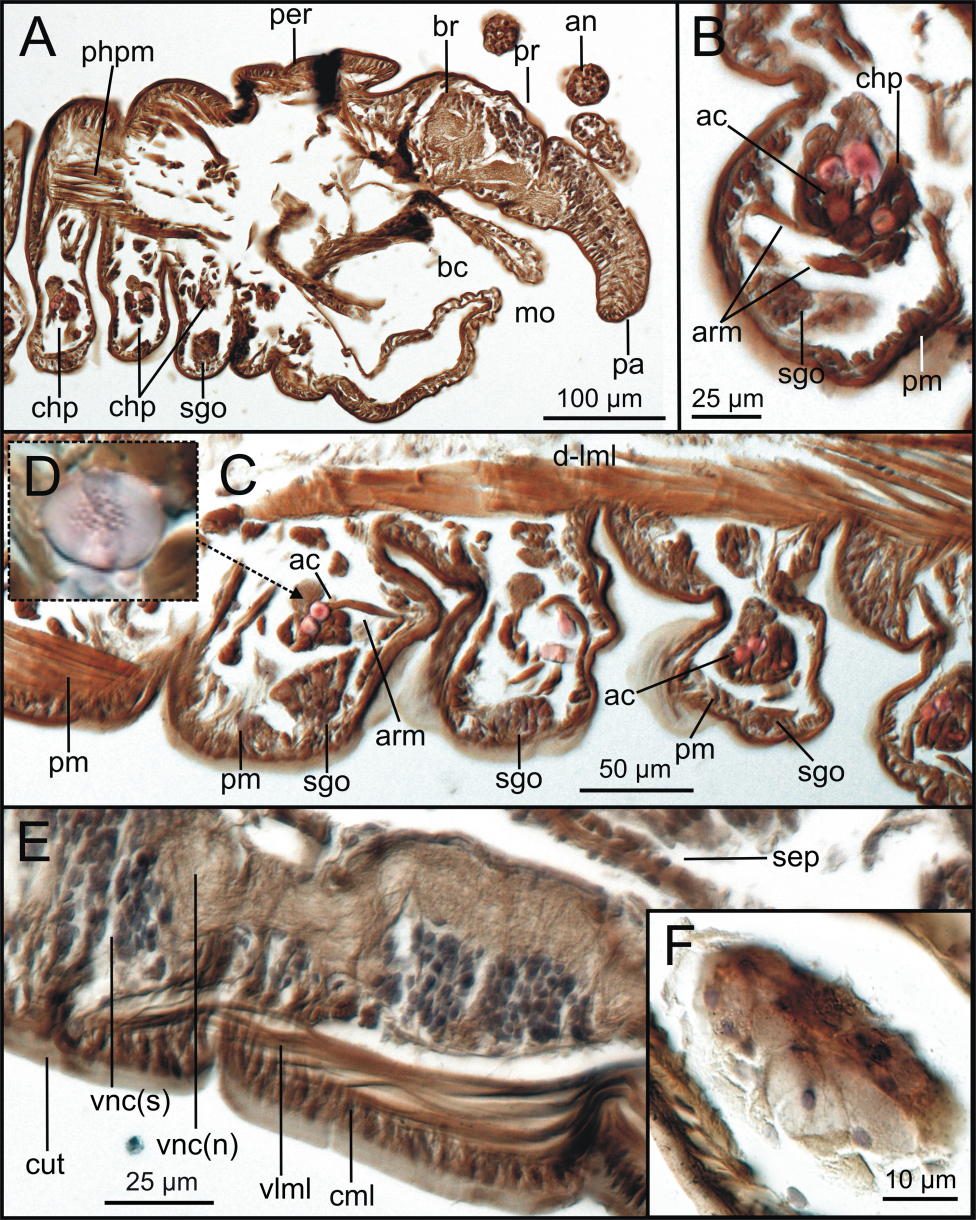
Sagittal histological sections of *Syllis gracilis*. One specimen stained with Hematoxylin-Eosin (MNCN 16.01/18368-18372). (A) Anterior body region showing prostomial brain, palp nerve, and segmental organ, located ventrally to parapodial aciculae in first anterior body segments; (B) detail of chaetae+aciculae group and segmental organ in ventral parapodia; (C) basal region of several parapodia showing segmental organs located ventrally to chaetal group; (D) detail of a transversally sectioned acicula showing internal chaetal channels; (E) detail of somata/neuropil regions of ventral nerve cord and elements of ventral body wall; (F) detail of segmental organ.

**Figure 10:**
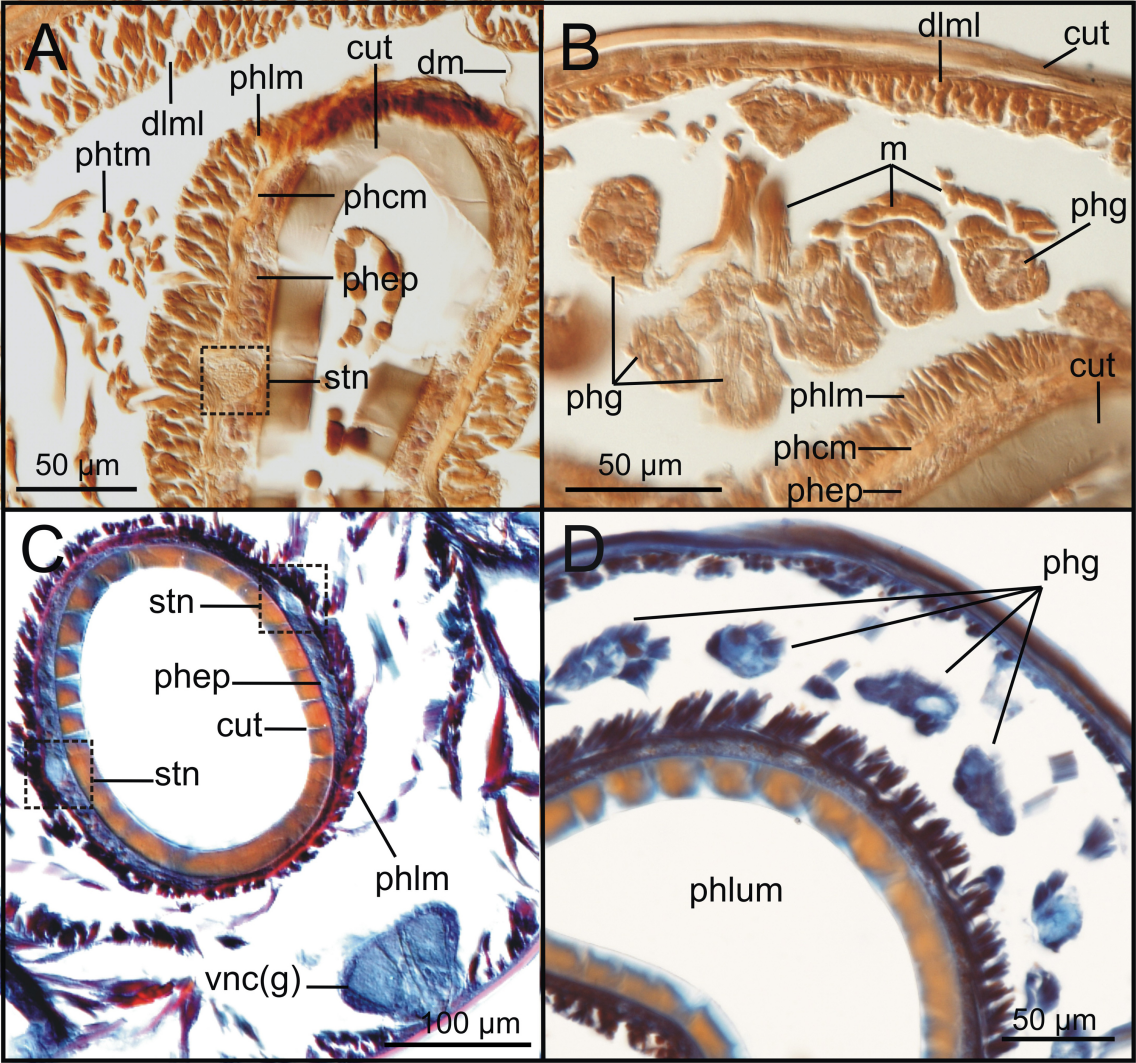
Transversal histological sections of *Syllis gracilis*. One specimen of stained with Hematoxylin-Eosin (MNCN 16.01/18342-18349) and Mallory (MNCN 16.01/18383-18398). (A, C) pharynx, showing a left stomatogastric nerve located in pharyngeal epithelium; (B) detail of antero-dorsal part of pharynx showing pharyngeal glands; (C) overall view of pharyngeal region showing main anatomical elements; framed: stomatogastric nerves at both sides of pharynx; (D) pharyngeal glands at dorsal side of pharynx.

**Figure 11:**
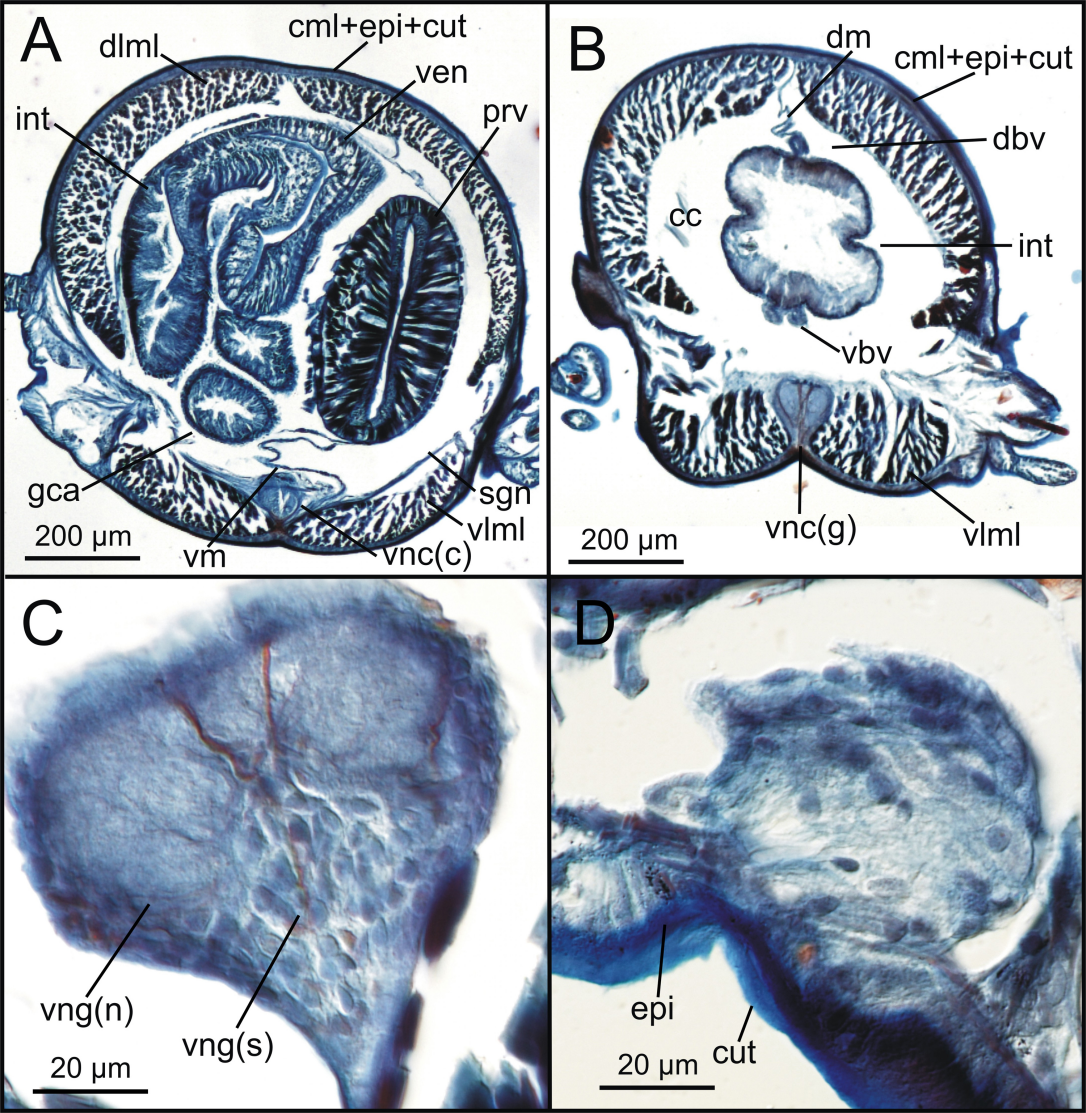
Transversal histological sections of *Syllis gracilis*. One specimen stained with Mallory (MNCN 16.01/18383-18398 and MNCN16.01/18399-18404). (A) posterior proventricle region showing connection with intestine and gastric caeca; (B) intestine and dorsal and ventral blood vessels; (C) detail of ventral cord ganglion showing soma and neuropil regions; (D) detail of one segmental organ at parapodium base.

The musculature of body wall, along with several internal organs (i.e., gut, see below), is among the features that are better-observed with the micro-CT, whether specimens are stained with iodine or otherwise. Body musculature is evident both in longitudinal—being coronal or sagittal (dlml, phpm, phrm, Figs. 5, 6)—and transversal body sections (Fig. 7). However, as stated above, it is difficult in all cases to detect the limits between wall muscles and the components of the integument and cuticle, which are far better portrayed in HIS images (Figs. 8–11). Three muscular elements are particularly well viewed through micro-CT: (1) the dorsal longitudinal muscular layer of body wall (dlml), (2), the muscles associated with parapodial movement, especially the acicular retractor muscles (arm), and (3) the protractor, retractor and transversal muscles associated with the pharyngeal movement (phrm, phpm, phtm). In contrast, the circular (or transversal) body wall muscular layer, which is much less developed than the longitudinal one, is more conspicuous in HIS (cml, Figs. 8E, 8E) than in micro-CT images (Fig. 7). Longitudinal musculature is evident in coronal (dlml, Fig. 6B), sagittal (Fig. 6A) and transversal (e.g., Figs. 7B, 7D) micro-CT sections. This appears as a large dorsal band mostly continuous across the body, the exception being at the pharyngeal level, where the transversal pharyngeal muscles are inserted in the inner side of body wall (phtm, Figs. 7B, 7C, arrowheads) (see below). This discontinuity in the longitudinal dorsal muscular bands results in a dorsolateral muscular band, that is located more ventrally (d-lml, Figs. 7C, 8C). On the ventral side of the body, there are two far less developed longitudinal muscular bands (vlml, Fig. 6A) located on both sides of the ventral nerve cord (vnc, Fig. 7D). In this case, once again, HIS shows greater detail both in the structure and limits of these muscular bands (Figs. 8C, 8D, 9E, 11A, 11B).

Muscular elements associated with parapodial and chaetal/acicular movement (arm) are reflected in far more detail in 3D micro-CT images (Figs. 5B, 6A, 7A–7D), even better than those of HIS (Figs. 8, 9).

Protractor, retractor and transversal pharyngeal muscles (phpm, phrm, phtm), associated with pharyngeal movement, are particularly well seen in micro-CT images (Figs. 5A, 6B, 7B), although the limits of each group of muscles are poorly defined. On the contrary, the circular and longitudinal musculature of the pharyngeal wall is better portrayed in HIS (phcm, phlm, Fig. 8C). The myotomes of the proventricle are radially disposed and can be clearly seen in all images (prv, Figs. 5A, 6B, 6A vs. 11A). Pharyngeal protractor muscles connect the anterior and posteriormost pharynx with the anterior body wall (phpm, Figs. 5A, 6B). Pharyngeal retractor muscles are located connecting the anterior end of the pharynx with the inner face of the body cavity backwards, which are difficult to identify among the pharyngeal transversal muscles (phrm, phtm, Figs. 5A, 6B). The latter are numerous and well defined, mostly in transversal sections of the pharynx; these muscles are arranged laterally on each body side, connecting the pharynx to the inner side of the body wall above the insertion of parapodial dorsal cirri (Fig. 7B), from about chaetiger 2 level to chaetiger 11, and then absent from the level of the proventricle (Fig. 7C).

Conversely, in the iodine-stained specimen, micro-CT images show muscular fibres, cuticle and epidermal elements that appear as if they are pasted to each other (Figs. 15C–15E).

### Coelomic cavity and vascular system

**Figure 12:**
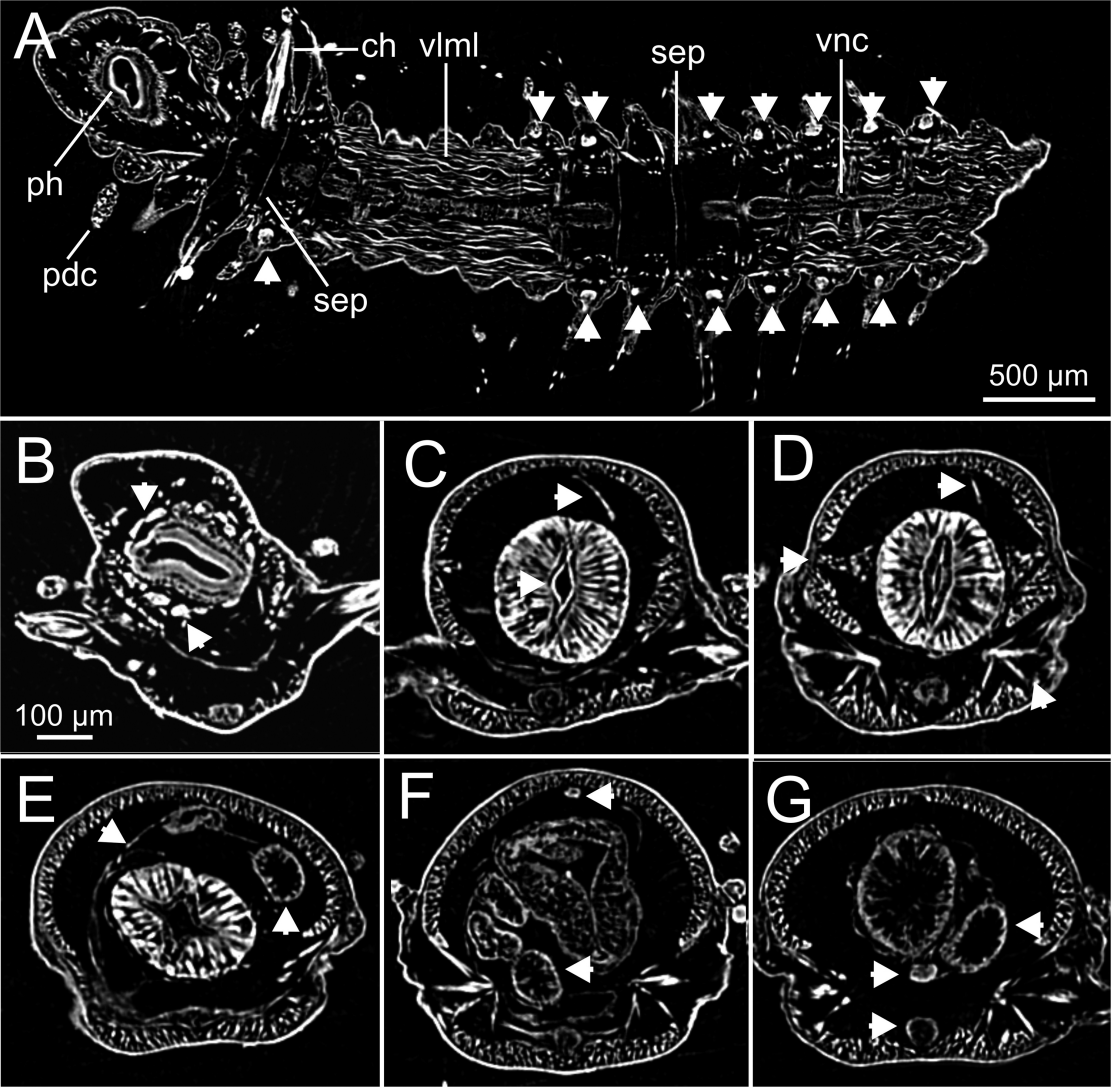
Micro-CT 2D images of internal anatomy of *Syllis gracilis*. Two unstained specimens (MNCN 16.01/18410 and MNCN16.01/18411). (A) hemifrontal section at ventral nerve cord level (arrowheads marking position of segmental organs); (B–G) several transversal sections at pharyngeal (B), anterior, middle, and posterior proventricle (C–E), ventricle (F) and intestine regions (G). Arrowheads marking several anatomical structures such as: dorsal and ventral pharyngeal glands in B; dorsal mesentery in C; dorsal mesentery (up), point of attachment of pharyngeal transversal muscles (left) and parapodial musculature complex (down, right) in D; peritoneal lining (left) and gastric caeca (right) in E; dorsal blood vessel (up) and gastric caeca (down) in F; gastric caeca (right), ventral blood vessel (middle), and ventral nerve ganglion (down) in G.

**Figure 13:**
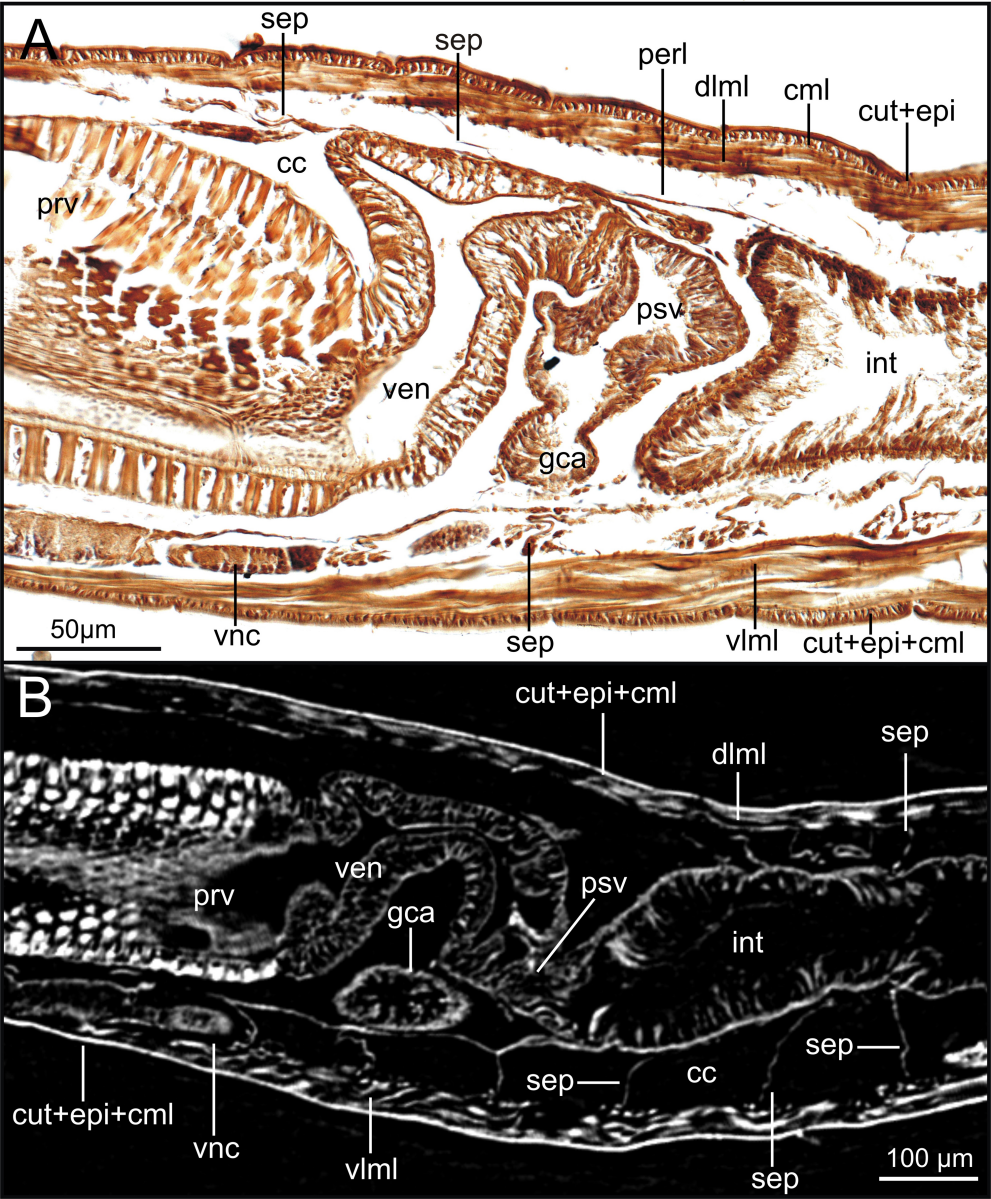
Comparison of images of proventricle-ventricle-intestine region of *Syllis gracilis*. Two specimens (MNCN 16.01/18368-18372 and MNCN 16.01/18410) as obtained from HIS (A) and virtual micro-CT 2D sagittal section (B).

The coelomic cavity and enclosed organs are clearly observed with both micro-CT and HIS (cc, Figs. 7, 8C, 11B). However, the peritoneum, the septa and the two major blood vessels are better defined in the micro-CT stained specimen, particularly in coronal and transversal (Figs. 15C, 15E) sections at the intestine level, as occurs with HIS (Fig. 11B). Micro-CT images of unstained specimens only show dorsal mesenteries and septa that are better seen in 2D sagittal sections (arrows, Fig. 12C, 12D; sep, Fig. 13B) than in 3D. Images of iodine-stained specimen show ventral peritoneum and its continuation towards the septa.

The major dorsal blood vessel is clearly seen in 3D micro-CT images, particularly in relation to the intestine, while the ventral vessel is only hardly discernible at that level (vbv, Fig. 7D). The micro-CT stained specimen shows far better results in this case. Both elements are better portrayed in HIS images of Mallory stained specimen (Fig. 11B).

There are neither discernible blood plexus nor lateral/segmental vessels, but in some 3D images of the ventricle-intestine region, a series of ‘lines’ that seem to represent thin segmental vessels connecting the two major longitudinal vessels were seen. However, the possibility of an artefact related to muscular fibres associated with the peritoneum cannot be ruled out.

**Figure 14:**
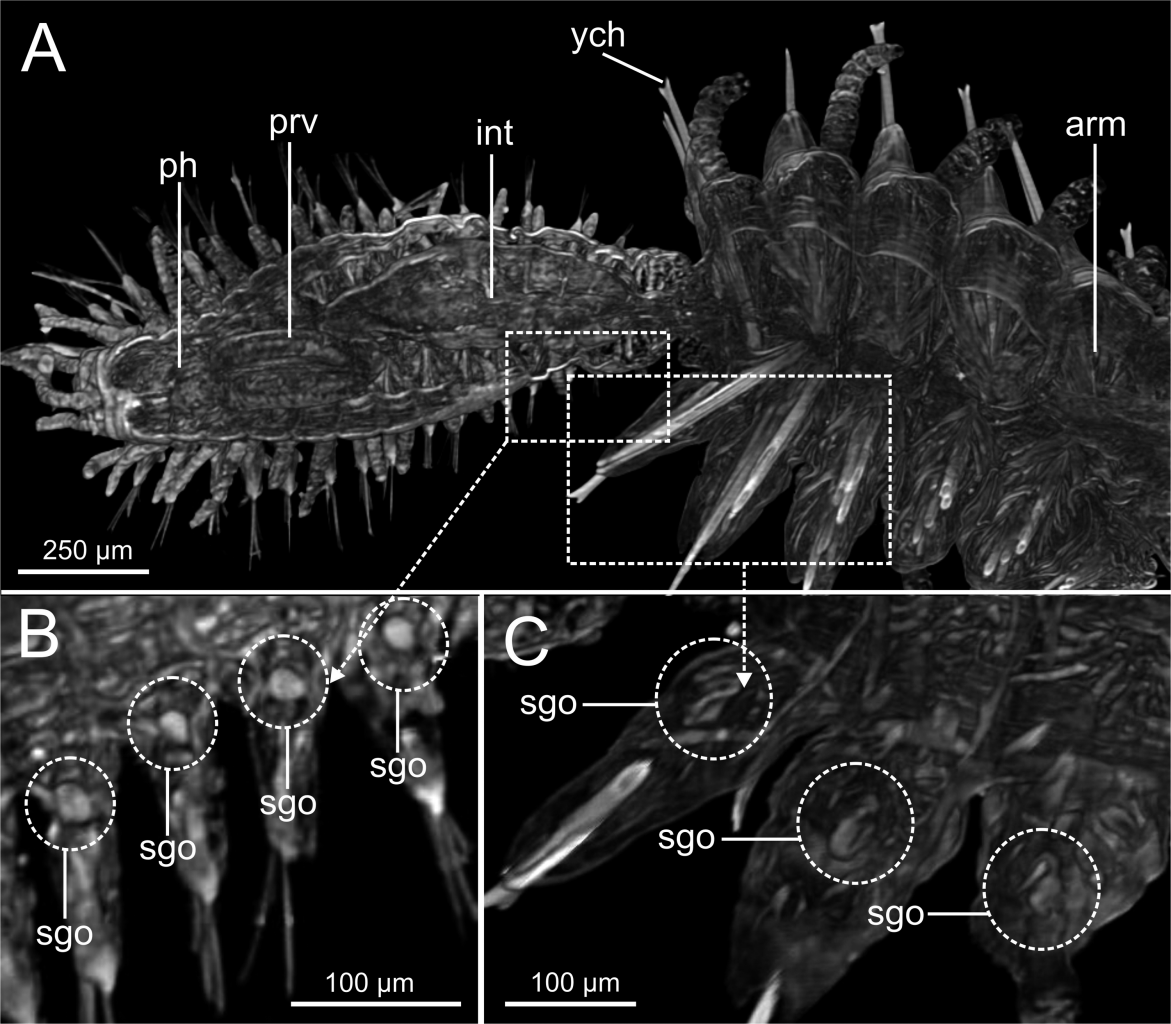
Micro-CT 3D volume renderings of internal anatomy of *Syllis gracilis*. One unstained specimen regenerating from a mid-body fragment (MNCN 16.01/18409). (A) coronal view; (B–C) detail of framed areas in A.

**Figure 15:**
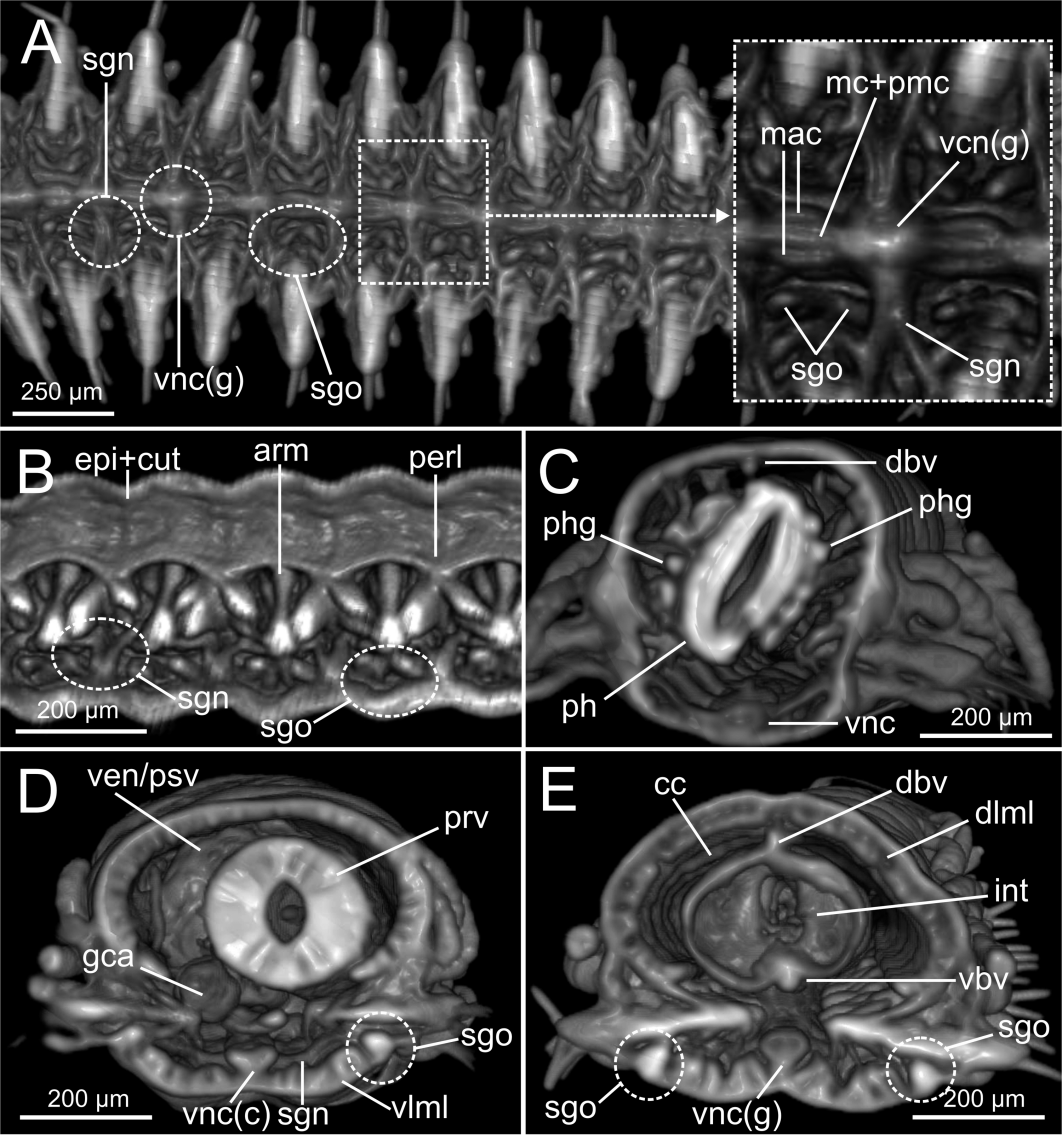
Micro-CT 3D volume renderings of internal anatomy of *Syllis gracilis*. Iodine-stained specimen (MNCN 16.01/18405). (A) Ventro-frontal section at intestine level showing ventral nerve cord, segmental nerves and segmental organs; framed: detail of ventral nerve cord; (B) right hemi-sagittal section showing organization of parapodial musculature and segmental organs; (C) transversal section at anterior pharyngeal region showing main body elements; (D) transversal section at middle proventricle region showing main body elements; (E) transversal section at intestine level showing main body elements.

### Digestive system

The foregut of *S. gracilis* is composed of an anterior eversible axial-type pharynx that is connected to the buccal opening through a pharyngeal sheath (phsh, Fig. 6B) and posteriorly leading to a muscular proventricle (ph, prv, Figs. 5A, 5B, 6A, 6B) provided with numerous well delineated muscle fibres that are radially disposed, and a posterior non-muscular ventricle (=oesophagus) provided with caeca (ven, gca, Figs. 5B, 6A). The midgut is made up of a long and apparently undifferentiated intestine (int, Figs. 5B, 6A) and the hindgut by a short undifferentiated rectum (not studied here).

Both 3D longitudinal and transversal micro-CT sections clearly reflect all gut regions and their inner anatomical composition. The anterior pharyngeal region is surrounded by thick pharyngeal glands (phg, Figs. 6B, 7A), also seen in 2D (Fig. 12B) and in HIS (Figs. 10B, 10D) and by numerous transversal and retractor muscles (phtm, phrm, Fig. 6B). The components of the pharyngeal wall, e.g., pharyngeal lumen, pharyngeal longitudinal muscles, and inner cuticular and epidermal lining, are clearly seen both in micro-CT (phlm, phep, phcm, Figs. 7A, 7B) and HIS (Figs. 8C, 10A, 10B) sections, but better in the latter, where a thin circular muscle lining (phcm, Fig. 10B) can also be much better discerned from the pharyngeal epithelium (phep, Fig. 10B).

The anterior and posterior parts of proventricle seem to show a similar shape throughout, both in lumen size and shape, and the radial disposition of the myotomes. The inner region of the anterior proventricle, at its junction with the pharynx, is lined with a cuticular plate, which is visible both in 3D (apcs, Figs. 5A, 7C) and 2D micro-CT images (Fig. 12C, arrowhead). Backwards, the ventricle (provided with gastric caeca) and the intestine can be observed (ven, gca, int, Figs. 5B, 6A), but they are composed of a far thinner wall than that of the pharynx and proventricle (Fig. 7D). In fact, 2D micro-CT (Figs. 12E–12G, 13B) and HIS (Figs. 11A, 11B, 13A) images show clear differences between these gut regions in relation to the epithelium; the ventricle epithelium is more glandular than that of the gastric caeca and intestine, which exhibit a much thinner wall, but have many long microvilli that are absent in the former (Fig. 13).

In contrast, micro-CT images taken from the iodine-stained specimen do not provide detailed information about these features in gut, showing only gross features at this level (Figs. 15C–15E).

### Excretory and reproductive systems

Masses with metameric disposition, here named as segmental organs (sgo), are clearly observed both in unstained 3D and 2D (Figs. 7B–7D, 12A, arrows), and stained micro-CT images (Figs. 15A, 15B, 15D, 15E), and as well in HIS (Figs. 8C, 9A–9C, 9F, 11D). These masses may correspond to metanephridia rather than to gonads when considering the non-reproductive external appearance of the specimens, their metameric disposition, their well-defined and constant size and shape along the body (Figs. 12A, 14C), and cell composition (Figs. 8C, 9F, 11D). Iodine-stained specimen shows conspicuous multiple, small cell masses at parapodial bases in the mid-anterior body segments (sgo, Figs. 15A, 15B), while a simple pair seems to be present in the posterior third (Figs. 15D, 15E). This suggests that evolving gonads may be present along with the nephridia in anterior segments but could represent an artefact corresponding to other structures such as parapodial muscle fibres, nerves or even circulatory vessels.

### Nervous system

The main elements of the annelid nervous system such as the brain and ventral nerve cord with associated ganglia are clearly observed in 2D and 3D micro-CT images, both in unstained (br, vnc (g), Fig. 7A), stained specimens (Figs. 15A, 15C, 15D) and in HIS (Figs. 8B–8D, 9A, 9E, 11A–11C). While the brain is much better viewed in HIS (br, Figs. 8B, 9A) than with micro-CT, the ventral nerve cord is one of the internal structures that is clearly shown with all techniques (vnc, e.g., Figs. 6A, 7A, 7C, 7D, 8C, 8D, 15A).

The internal organization of ventral ganglia (i.e., central core of neuropil and a peripheral ring containing neuronal somata (vng (s), vng (n), Figs. 8D, 11C) is also present in HIS and micro-CT images, but less clearly in the latter (cfr. Figs. 8C, 11B vs. 15C, 15D, 15E). Most neurons are located mid-ventrally and laterally in ventral ganglia, but dorsally and laterally in the brain (br(n), br(s), Fig. 8B). The distinct location of neuron bodies and axons (somata and neuropil respectively) in brain and segmental ganglia is, undoubtedly, much better shown in HIS images. In contrast, the micro-CT images show these features with less detail both in unstained 3D (Fig. 7D) and stained (Fig. 15D) specimens. Nevertheless, micro-CT fronto-ventral images of stained specimen show with considerable detail the multiple fibres that run between the ventral nerve cord ganglia (Fig. 15A, insert) that seem to correspond to the multiple, partially fused, connective fibres present between each segmental ganglion.

In relation to the peripheral nervous system, segmental nerves emanating from the ventral cords are also clearly shown in these images, where a pair of segmental nerves runs from each ganglion of the ventral nerve cord (sgn, Fig. 15A). In addition, thin nerves in palps, antennae and parapodial cirri are only detected with HIS (pan, man, tdcn, pcn(b/p), Figs. 8A, 8B, 8E), as well as the circumoesophageal connectives that connect the brain to the ventral nerve cord (coec, Fig. 8B).

The stomatogastric complex can only be seen in HIS transversal sections at pharyngeal level with both staining methods (stn, Figs. 10A, 10C), but not with micro-CT in the same sections (Figs. 7A, 7B), neither in 2D and 3D renderings nor in unstained or iodine-stained specimens.

In relation to the sensory system, HIS shows prostomial eyes (eye, Fig. 8B) and SEM images highlight the position of the nuchal organs (nuo, Fig. 4B), the latter being poorly distinguished in micro-CT images.

## Discussion

### Previous anatomical studies in the genus *Syllis*

The overall anatomy of the genus *Syllis* is well known since the 19th century (e.g., Audouin & Milne-Edwards, 1833; Grube, 1840; Delle Chiaje, 1841–1844 and Rathke, 1843). Some internal characters (shape and size of anterior gut) were then used as diagnostic character at the species level. The internal syllid anatomy was later studied by many authors, and recently by Aguado et al. (2015), Helm & Capa (2015) and Weidhase et al. (2016), Weidhase et al. (2017), using HIS and CLSM. Recently, Faulwetter et al. (2013a), Faulwetter et al. (2013b) and Parapar et al. (2017a) provided the first micro-CT images of the anatomy of *S. gracilis* and *Syllis garciai* (Campoy, 1982) respectively; however, unlike in our study, these authors examined just one specimen and only described gross elements of external and internal anatomy such as gut (pharynx, proventricle) and body musculature (parietal longitudinal bands), not comparing these results with any other anatomical techniques. Unfortunately, the brief description of the main body elements in the Mediterranean specimen of *S. gracilis* provided by Faulwetter et al. (2013a) and Faulwetter et al. (2013b), does not allow for an accurate comparison with our specimens. However, the description of *S. garciai* provided by Parapar et al. (2017a) allowed us to highlight potential differences that may be relevant for future taxonomic/phylogenetic studies:

(1) The circulatory system seems to be more complex in *S. garciai* than in *S. gracilis*, showing well developed lateral blood vessels joining dorsal to ventral blood vessels in the former through well-developed segmentary vessels.

(2) The shape of the proventricle lumen of *S. garciai* changes longitudinally, being anteriorly circular in cross section and star-shaped posteriorly. This change in shape was not detected in any examined specimen of *S. gracilis*. Nevertheless, according to Parapar et al. (2017a), this could be an artefact due to the preservation process or post manipulation of the only studied specimen. In any case, we consider that this aspect is worthy of further study by examining more specimens, and thus potentially revealing additional interspecific differences.

### External anatomy and taxonomy

Compared to clitellate annelids (see Fernández et al., 2014), the taxonomy of “polychaetes”, and especially for syllids, mostly relies on external rather than on internal characters (e.g., Álvarez Campos et al., 2015).

The examination of many specimens of *S. gracilis* in this study, by means of micro-CT, provided good images of several relevant characters critical for this species, such as the shape, length and number of articles of dorsal parapodial cirri, but not of the same quality and reliability as those obtained by using the traditional imaging tools such as the STM, LCM and SEM. For instance, the morphology of posterior compound chaetae and the degree of fusion of blades and shafts in the ypsiloid chaetae are much better depicted by SEM, while the colouration pattern (see Álvarez Campos, Giribet & Riesgo, 2017) is only properly visualized with STM.

### Internal anatomy

The integrative approach of several techniques has provided a full view of the internal anatomy of *S. gracilis*, also allowing for a comparison of their performance. In this case, the micro-CT showed its full potential and allowed us to visualize, in 3D, the natural spatial arrangement of the different organs, without affecting the specimens’ integrity.

### Integument and musculature

Polychaete integument consists of a monolayered epithelium containing several types of cells, covered by a cuticle and lying upon a thin fibrous extracellular layer (Storch, 1968). The Syllidae have a special kind of integument related to beaded antennae and cirri that are provided with a central core of musculature and nervous tissue, surrounded by several types of integumental cells, including cork-screw shaped long and slender gland cells (Storch, 1968). These characters are relevant in *Syllis* taxonomy and included in many descriptions of *S. gracilis* such as San Martín (2003) and Álvarez Campos et al. (2015). All these cirri components are evident in the *S. gracilis* specimens studied with both LCM and HIS, but were poorly viewed with micro-CT.

The main elements of polychaete musculature are the circular (transversal) and longitudinal fibres of the body wall, parapodial, chaetal, oblique, diagonal, and dorsoventral fibres, as well as muscular elements related to septa and mesenteries (Tzetlin & Filippova, 2005). Both HIS and micro-CT images of the studied specimens closely reflect the organization of the dorsal muscle band, as described by these authors, consisting of up to 10 or more muscle bundles adjacent to each other, plus two ventral bands. The poorly developed circular layer seen in these studies agrees with previous works on other annelid families. However, some areas of the body wall of *S. gracilis* appear to have a thin layer of this kind of muscular fibres, which is more evident in HIS images than in micro-CT ones, thus confirming once again what has been previously reported for other syllid species (Mettam, 1967; Mettam, 1971; Tzetlin & Filippova, 2005; Purschke & Müller, 2006; Filippova et al., 2010; Helm et al., 2013; Helm & Capa, 2015). Muscular fibres of the parapodial muscle complex are always well developed in vagile parapodial bearing species, as is the case of *S. gracilis*, and consists of numerous muscles associated with the parapodial wall, intrinsic muscles located inside the parapodium, diagonal and transversal (circular) muscles and muscles attached to aciculae and compound chaetae (Storch, 1968; Tzetlin & Filippova, 2005; Helm et al., 2013; Helm & Capa, 2015). Unfortunately, micro-CT does not provide images with sufficient resolution to detect many of these muscular bundles, especially the thinner ones, showing only those with a larger diameter (i.e., acicular protractor muscles). Only HIS techniques and CLSM methods, such as those used in Aguado et al. (2015) and Helm & Capa (2015), can provide better information on these bundles.

### Coelomic cavity and vascular system

Syllids have a well-developed fluid-filled coelomic space between the body wall and the gut wall. The coelomic peritoneum, which defines the dorsal and ventral mesenteries that suspend the gut within the body, encloses a pair of inner spaces in each segment and surrounds the two major, dorsal and ventral, blood vessels. An exception occurs in the foregut, which is involved in the extensive longitudinal movements of the proboscis (Gardiner, 1992). *Syllis gracilis* shows a wide coelomic cavity, which was also previously reported by Faulwetter et al. (2013b) for this species, and Parapar et al. (2017a) for *S. garciai*. We have noted that the coelom is divided by septa even from the proventricle region, although this division may be incomplete there; these septa were particularly well seen in sagittal 2D and 3D micro-CT sections of both unstained and stained specimens.

The circulatory system of *S. gracilis* seems less complex than that of other annelid families, which show highly muscularized vessels (heart bodies), a sinus or plexus associated with the gut, and a wide variety of perineural, segmental or head vessels (see Malaquin, 1893; Gardiner, 1992; Pleijel, 2001). Furthermore, when considering the findings by Parapar et al. (2017a) for *S. garciai*, it seems that further interspecific differences may occur. Thus, the circulatory system of *S. gracilis* seems to lack the well-developed pair of longitudinal lateral vessels that are located on both sides of the gut of *S. garciai*. A closer examination using more specimens of *S. garciai* is needed to confirm the presence of such a feature.

### Digestive system

Even though Syllidae show a homonomously segmented body, the alimentary canal is highly regionalized and shows different specialized parts (see Tzetlin & Purschke, 2005; San Martín & Aguado, 2014); these are mainly located in the ectodermic foregut that is divided into a short pharyngeal sheath, a long pharyngeal tube, a conspicuous muscular proventricle and a thin-walled ventricle, provided with a pair of lateral caeca (Jenniaux, 1969; Glasby, 2000). All these elements are clearly observed by all the techniques used in this study. Particularly, the micro-CT clearly shows even the minute anatomical details of this region, such as the thin pharyngeal sheath—usually not illustrated in most traditional taxonomic representations of *Syllis* species (e.g., Fig. 1A in this work and Álvarez Campos et al., 2015)—the radial disposition of the muscular myotomes and the cuticular plates located at the anterior end of the proventricle, described in detail by Boilly (1967) and Michel (1974) that might assist in the mechanical digestion of food (Haswell, 1921). Observed differences in the inner epithelium composition of each part, far more ciliated in caeca and endodermic midgut (intestine) than in the ventricle, was previously reported by Jenniaux (1969) and Glasby (2000).

All these gross gut elements were also reported by Faulwetter et al. (2013b) and Parapar et al. (2017a); however, Faulwetter et al. (2013b) did not describe this aspect in detail for *S. gracilis* to allow for an appropriate comparison with our specimens. On the other hand, the main difference between the gut of *S. garciai sensu* Parapar et al. (2017a) and *S. gracilis* as is described here, lies in the shape of the proventricle lumen. Parapar et al. (2017a) describe a star-shaped lumen in the anterior half, while that of *S. gracilis* is constant along its full length. These authors consider that this change of shape may represent an artefact due to the state of preservation of the only examined specimen. However, we suggest that this potential difference deserves to be tested on more specimens of *S. garciai* as well as in other species, to assess whether it represents a useful taxonomic character.

### Excretory and reproductive systems

Excretory and reproductive systems in annelids are closely related and show great diversity across taxa. Thus, in Clitellata the nephridia are usually segmentally positioned, and gonads appear in a few, well defined segments. On the contrary, polychaetes show segmentary gonads that can be associated with nephridia in different body regions such as intersegmental septa, ventral or nephridial blood vessels, blind-ended capillaries, parapodial connective tissue and coelomic peritoneum (Glasby, 2000; Rouse & Pleijel, 2001). According to Goodrich (1945), the excretory ducts (metanephridia) and gonoducts (coelomoducts) of Syllidae are fused to each other, forming the metanephromixia. However, Bartolomaeus (1999) disputes their multiple origin. Whether they are derived from one or two different tissues, these body elements are disposed metamerically, numbering one pair per segment; they serve for both excretion and gamete emission and are named ‘segmental organs’ by Rouse & Pleijel (2001), a terminology followed by us here.

The Syllinae show a unique reproductive strategy called schizogamy, which is characterised by the production of stolons that are subsequently budded off and become pelagic (Garwood, 1991; Franke, 1999; Pleijel, 2001). In many polychaetes, the gametes are usually expelled from the body through metameric gonoducts associated with nephridia. In epitokous taxa, such as *S. gracilis*, only the stolons are thought to develop gonads. However, it has been suggested that in several Syllinae species the gametes are formed in the medium/posterior segments during the development of stolons and are then moved into the stolon through septal openings (Wissocq, 1966; Aguado et al., 2015).

Both HIS and micro-CT 2D and 3D images obtained here, from unstained and stained small and medium-sized specimens, show segmentally arranged discrete masses (segmental organs) located at the base of the parapodia, and along almost the entire body, in the same position in all individuals; these characteristics point to a metanephridial nature. Fragments of large-sized individuals regenerating the anterior end, also studied here (see Fig. 14), show these masses as coiled tubes and, therefore, also suggesting their metanephridial rather than gonadal nature. However, examination of the iodine-stained specimen shows multiple masses in anterior segments that would suggest that a different element, gonads or even artefacts created by other body elements, may be involved. Future studies of mature individuals in early schizogamic process would help to test this possibility.

### Nervous system

The central nervous system in Annelida is composed of an anterior prostomial brain and a ventral nerve cord (VNC), provided with a pair of segmentally arranged ganglia, and both connected by circumesophageal or circumpharyngeal nerves (Orrhage & Müller, 2005). The presence of both elements can be seen in both micro-CT and HIS images, although far better delineated in the case of the VNC. The micro-CT 3D images of stained/unstained specimens are far better detailed than those provided by Faulwetter et al. (2013a), Faulwetter et al. (2013b) and Parapar et al. (2017a).

The 3D reconstruction of the ventral nerve cord of the stained specimen agrees well with the organization proposed by Müller (2006) and Weidhase et al. (2017) for *Typosyllis antoni* Aguado et al., 2015. Thus, there is a triple interganglia connective whose central fibre may correspond to the median and paramedian connective *sensu* Weidhase et al. (2017); the two lateral fibres may correspond, in turn, to the main connective (compare with Fig. 7 in Weidhase et al., 2017). Weidhase et al. (2017) state that VNC communicates with the periphery via segmental nerves, usually three or four pairs per segment, and in many errant polychaetes, a pair of peripheral parapodial ganglia are present in each segment laying on segmental nerve II. Again, images of the stained specimen conspicuously show these segmental nerves projecting from the paired ganglia of the VNC. We did, however, not detect any segmental ganglia at the base of each parapodium of this species, as was reported by Parapar et al. (2017a) for the phyllodocid *Phyllodoce lineata* (Claparède, 1870).

### Micro-CT vs. HIS, CLSM and TEM

Many polychaete descriptions are traditionally based on stereomicroscopy, frequently supported by whole body optical microscopy and SEM, while micro-dissection and histology has normally been applied for purely anatomical studies (see above). However, the use of micro-CT for species identification in other taxa seems particularly relevant since it avoids specimen destruction, which is inevitable after histological sectioning (Fernández et al., 2014; Pedrouzo et al., 2017; Machado, Passos & Giribet, 2018). This is an evident advantage of micro-CT over HIS, although the former has obvious limitations to properly visualize many characters, especially at cellular level and, therefore, classical histological techniques are still essential for descriptions of new species when a proper identification of internal structures is required.

Traditional dissection is also widely used for the study of the internal anatomy in several groups of annelids, as is the case of the earthworms and large-sized polychaetes such as Terebellidae (e.g., Jouin-Toulmond, Agustin & Toulmond, 1996; Zhadan & Tzetlin, 2003; Parapar & Hutchings, 2015). However, this procedure is unsuitable for all small-sized polychaetes, as is the case of the Syllidae.

Another relevant aspect of the use of micro-CT, when considered as a virtual dissection technique, is that it allows for the examination of internal organs, in their natural anatomical context, without harming body integrity. Furthermore, by using appropriate software, the stack of data can be stored and shared with other scientists (see Keklikoglou et al., 2016), thus allowing for a far better and quicker dissemination and exchange of information.

On the other hand, Confocal Laser Scanning Microscope (CLSM) is becoming an essential tool in the study of invertebrate anatomy in general, and of annelids and syllids in particular (e.g., Hessling & Westheide, 1999; Hessling & Purschke, 2000; Müller & Westheide, 2000; Aguado et al., 2015; Helm & Capa, 2015; Weidhase et al., 2016; Weidhase et al., 2017). Here, our approach with this technique was focused on external anatomy because results can be easily compared with STM, LCM and SEM. In contrast, the internal anatomy of *S. gracilis* was not studied either with CLSM or Transmission Electron Microscopy (TEM) because resulting images at cellular level are not comparable with those provided by the micro-CT and, therefore, beyond the scope of this study.

### Micro-CT 2D vs. 3D images, staining vs. unstaining, artefacts

As stated by Parapar et al. (2017a), micro-CT 2D images in polychaetes are usually less informative than 3D renderings in relation to organ structure and position in body cavity but, in turn, they offer a complementary point of view. In HIS pictures, as seen with 2D images, some delicate structures are better visualized than in 3D, as is the case of the segmentary septa and inner ciliated lining of the intestine, both well seen in sagittal (cfr. Fig. 13) and coronal sections. In fact, we mostly agree with Fernández et al. (2014) who state that by combining 2D and 3D dataset visualization techniques, many morphological features commonly used in taxonomy can now be analysed without the need for dissection.

Although X-rays used in micro-CT scanning are particularly suitable for showing highly mineralized elements, electron-dense contrast agents such as iodine can be used for enhancing the contrast of soft, low dense, body tissues. Many staining strategies for soft-bodied invertebrates in *micro-* CT applications have been evaluated in several annelid taxa such as hirudineans (e.g., Tessler et al., 2016; Cascarano et al., 2017), oligochaetes (Fernández et al., 2014) and polychaetes (Paterson et al., 2014). In our experience, staining agents such as iodine can greatly improve visualization of tissues that are sometimes difficult to see in unstained specimens. Internal structures such as the different elements of the ventral nerve cord, the coelomic lining and the circulatory vessels can be much better observed, although this can result in these stained specimens in a ‘pasted’ appearance of the other denser organs/systems such as the digestive and the body musculature. Besides, although the term ‘non-invasive’ is usually used in relation to the use of micro-CT (Fernández et al., 2014), staining of specimens can be considered as an invasive technique because the used solutions cannot always be fully washed out from the sample after scanning.

According to our experience and as reported in previous works (e.g., Fernández et al., 2014; Parapar et al., 2017a), interpretation of micro-CT images should always be aware of the potential presence of artefacts. When using micro-CT and SEM, the presence of such elements (of very different origin) can lead to erroneous observations. For example, two specimens scanned the same day with same parameters, but collected at different dates and probably in different conditions, led to very different results in terms of image definition (cfr. Figs. 7A vs. 7B). Similarly, the relatively slow diffusion rate of the chemical staining agents (Fernández et al., 2014) or incomplete development of some structures, confirmed here in relation to the segmental organs (cfr. Figs. 14 and 15) can result in images that may lead to confusion. Therefore, multiple specimens should ideally be scanned to minimize the risk of obtaining wrong data from a single, possibly unsuitable specimen.

## Conclusions

•The integrative use of different anatomical techniques for the study of the anatomy of the marine annelid *Syllis gracilis* Grube, 1840 has confirmed previous findings in other annelids. Regarding micro-CT, although it needs proper infrastructure and qualified personnel, as occurs with HIS and SEM as well, the specimens can be relatively easily and quickly prepared and scanned, body integrity is not substantially altered, and the same specimen can be later subjected to other techniques (e.g., HIS, SEM and CLSM).

•Images shown here were obtained from specimens that were fixed in the usual manner, many specimens are stored in collections across the world and, therefore, do not follow the most suitable protocol for any given technique. This avoids obtaining top quality images but can help to evaluate their quality when comparing.

•Main external anatomical elements of *S. gracilis* are shown with all techniques, but micro-CT images show lesser optical resolution and contrast when compared to micrographs provided by LCM, SEM and CLSM.

•Comparison of micro-CT and HIS images revealed the utility and reliability of the former to show the presence, shape and spatial disposition of most internal body organs; the resolution of micro-CT images at cellular level is, however, much lower than that of HIS, which makes both techniques complementary.

•Main internal anatomical elements of *S. gracilis* are clearly portrayed in micro-CT images, but however, the best visualized features are those of higher tissue density (i.e., musculature, gut), particularly in 3D images of unstained specimens, while images of less electron dense tissues (i.e., peritoneal lining of septa and nervous system) lack sufficient detail. For the latter, 2D imaging and specimens staining works better.

•One of the main advantages of the micro-CT technology is that the anatomical structures are observable in their natural position; this may potentially be useful for taxonomic (and phylogenetic) studies and thus complementing traditional characters, that in Syllidae comprises mainly external structures currently examined by LCM and SEM.

•The use of iodine stain with micro-CT has shown advantages over non-staining for the adequate observation of delicate elements of low density, showing spectacular images of the segmental organs as well as the connective between the ganglia, the ventral nerve cord and segmental nerves. On the contrary, images lack much detail for high density elements (e.g., musculature, digestive tract).

•Scanning artefacts and, consequently, misinterpretation of structures can be avoided by simultaneously using other techniques (e.g., HIS) and examination of multiple specimens at varying stages of development.

### Abbreviations

ac: acicula
a. e.: anterior end
an: antenna
apcs: anterior proventricle cuticle sheath
arm: acicular retractor muscles
bc: buccal cavity
bl: buccal lip
bms: blade marginal spines
br: brain
br(n/s): brain (neuropil/somata)
cart: cirrus articles
cc: coelomic cavity
cfch: compound falciger chaetae
ch: chaetae
chb: chaetal blade
chm: chaetal muscles
chp: chaetal protractor muscles
chs: chaetal shaft
CLSM: confocal laser scanning microscopy
cml: circular muscle layer
coec: circumoesophageal connective
cut: cuticle
D: drawings
dbv: dorsal blood vessel
dlml: dorsal longitudinal muscle layer
d-lml: dorso-lateral longitudinal muscle layer
dm: dorsal mesentery
dst: distal tooth
epi: epidermis
eye: eyes
fl: fusion line
gca: gastric caeca
HIS: histological sections
int: intestine
la: lateral antenna
LCM: light compound microscopy
m: muscles
ma: median antenna
mac: main connective
man: median antennal nerve
mc: median connective
micro-CT: micro-computed X-ray tomography
mo: mouth
ms: marginal spines
nuo: nuchal organ
pa: palp
pan: palp nerve
par: parapodium
pcg: parapodial cirri glands
pcm: parapodial cirrus musculature
pcn (b/p): parapodial cirrus nerve (body/processes)
pcul: pharyngeal cuticular lining
pdc: parapodial dorsal cirrus
per: peristomium
perl: peritoneal lining
ph: pharynx
phcm: pharyngeal circular muscles
phep: pharyngeal epithelium
phg: pharyngeal glands
phlm: pharyngeal longitudinal muscles
phlum: pharyngeal lumen
phpa: pharyngeal papillae
phpm: pharyngeal protractor muscles
phrm: pharyngeal retractor muscles
phsh: pharyngeal sheath
pht: pharyngeal tooth
phtm: pharyngeal transversal muscles
pm: parapodial muscles
pmc: paramedian connective
pr: prostomium
prt: proximal tooth
prv: proventricle
prvmf: proventricle muscular fibres
psv: postventricle
pvc: parapodial ventral cirrus
SEM: scanning electron microscopy
sep: septum
sgn: segmental nerve
sgo: segmental organ
ss: shaft spines
STM: stereomicroscopy
stn: stomatogastric nerves
tc: tentacular cirrus
tdc: tentacular dorsal cirrus
tdcn: tentacular dorsal cirrus nerve
tvc: tentacular ventral cirrus
vbv: ventral blood vessel
ven: ventricle
vlml: ventral longitudinal muscle layer
vm: ventral mesentery
vnc: ventral nerve cord
vnc(g/c): ventral nerve cord (ganglium/connective)
vnc (n/s): ventral nerve cord (neuropilum/somata)
vng(n/s): ventral nerve ganglium (neuropilum/somata)
w. a.: whole animal
ych: ypsiloid chaeta
yms: ypsiloid marginal spines

##  Supplemental Information

10.7717/peerj.7251/supp-1 Table S1 Features of the sampling localities Biotic and abiotic data of the localities where the *Syllis gracilis* specimens were collected.

10.7717/peerj.7251/supp-2 Table S2 CLSM treatment Preparatory treatment of *Syllis gracilis* specimens examined with CLSM.

10.7717/peerj.7251/supp-3 Table S3 Histology treatment Preparatory treatment of histological sectioned specimens of* Syllis gracilis*.

10.7717/peerj.7251/supp-4 Table S4 Micro-CT treatment Preparatory treatment and scanning parameters of micro-CT studied specimens of *Syllis gracilis*.

## Additional Information and Declarations

### Data Availability

The following information was supplied regarding data availability: The examined specimens are available at http://www.mncn.csic.es/ in the Museo Nacional de Ciencias Naturales, Madrid (MNCN) repository via accession numbers MNCN 16.01/18341 to MNCN16.01/18415.
