# Supplementary material for: An integrative approach to the anatomy of Syllis gracilis Grube, 1840 (Annelida) using micro-computed X-ray tomography
Source: PeerJ. 2019 Jul 8;7:e7251. doi: 10.7717/peerj.7251 (PMC6622173; doi:10.7717/peerj.7251)
Supplement: Table S1 — Biotic and abiotic data of the localities where the Syllis gracilis specimens were collected. [file peerj-07-7251-s001.docx]

| **TABLE 1** |  |  |  |  |  |  |  |  |  |
| --- | --- | --- | --- | --- | --- | --- | --- | --- | --- |
|  | **Museum reference** | **Extracted from** | **Figure/s** | **Locality** | **Habitat** | **Sampling date** | **Latitude (N)** | **Longitude (W)** | **Depth (m)** |
| **D** | MNCN 16.01/18405 | MNCN 16.01/16001 | 1A-E | Fornelos | crevice top | 7-apr-01 | 43º28'04.68'' | 008º18'47.17'' | rocky intertidal |
| **STM** | MNCN 16.01/18342-18349 | MNCN 16.01/15999 | --- | Fornelos | crevice top | 26-oct-00 | 43º28'04.68'' | 008º18'47.17'' | rocky intertidal |
|  | MNCN 16.01/18368-18372 | MNCN 16.01/15999 | --- | Fornelos | crevice top | 26-oct-00 | 43º28'04.68'' | 008º18'47.17'' | rocky intertidal |
| **LCM** | MNCN 16.01/18350-18367 | MNCN 16.01/15999 | 2A, B | Fornelos | crevice top | 26-oct-00 | 43º28'04.68'' | 008º18'47.17'' | rocky intertidal |
|  | MNCN 16.01/18368-18372 | MNCN 16.01/15999 | 2C-F | Fornelos | crevice top | 26-oct-00 | 43º28'04.68'' | 008º18'47.17'' | rocky intertidal |
| **SEM** | MNCN 16.01/18341 | MNCN 16.01/15999 | 4A-G | Fornelos | crevice top | 26-oct-00 | 43º28'04.68'' | 008º18'47.17'' | rocky intertidal |
| **HIS** | MNCN 16.01/18342-18349 | MNCN 16.01/15999 | 8C, E, 10A, B | Fornelos | crevice top | 26-oct-00 | 43º28'04.68'' | 008º18'47.17'' | rocky intertidal |
|  | MNCN 16.01/18350-18367 | MNCN 16.01/15999 | 8A, B, D | Fornelos | crevice top | 26-oct-00 | 43º27'41.70'' | 008º18'47.17'' | rocky intertidal |
|  | MNCN 16.01/18368-18372 | MNCN 16.01/15999 | 9A-F, 13A | Fornelos | crevice top | 26-oct-00 | 43º27'41.70'' | 008º18'47.17'' | rocky intertidal |
|  | MNCN 16.01/18373-18382 | MNCN 16.01/15999 | --- | Fornelos | crevice top | 26-oct-00 | 43º28'04.68'' | 008º18'47.17'' | rocky intertidal |
|  | MNCN 16.01/18383-18398 | MNCN 16.01/16011 | 11A-D | A Redonda | *Fucus vesiculosus* | 07-may-01 | 43º27'46.32'' | 008º16'12.81'' | rocky intertidal |
|  | MNCN 16.01/18399-18404 | MNCN 16.01/16013 | 10C, D | Laxe | *Fucus serratus* | 16-nov-01 | 43º27'52.26'' | 008º17'04.20'' | rocky intertidal |
| **micro-CT** | MNCN 16.01/18405 | MNCN 16.01/16001 | 15A-E | Fornelos | crevice top | 7-apr-01 | 43º28'04.68'' | 008º18'47.17'' | rocky intertidal |
|  | MNCN 16.01/18406 | MNCN 16.01/16003 | --- | Caleira | *Fucus vesiculosus + Mytilus galloprovincialis* | 26-may-02 | 43º29'28.64'' | 008º11'12.04'' | rocky intertidal |
|  | MNCN 16.01/18407 | MNCN 16.01/16007 | --- | Caranza | *Fucus serratus* | 26-may-02 | 43º28'55.73'' | 008º12'30.17'' | rocky intertidal |
|  | MNCN 16.01/18408 | MNCN 16.01/16012 | --- | Caranza | *Fucus vesiculosus* | 23-aug-02 | 43º28'55.73'' | 008º12'30.17'' | rocky intertidal |
|  | MNCN 16.01/18409 | MNCN 16.01/16000 | 14A-C | Fornelos | *Chondrus crispus* | 26-oct-00 | 43º28'04.68'' | 008º18'47.17'' | rocky intertidal |
|  | MNCN 16.01/18410 | ---- | 6B, 7A, 12B, F, 13B | Santa Lucía | mud | 12-dec-08 | 43º27'42.30'' | 008º14'35.98'' | 2.24 |
|  | MNCN 16.01/18411 | ---- | 5A, B, 6A, 7B, C-D, 12A, C-E, G | Santa Lucía | mud | 26-jun-15 | 43º27'41.70'' | 008º14'32.40'' | 1.16 |
| **CLSM** | MNCN 16.01/18412 | MNCN 16.01/16001 | 3E-F | Fornelos | crevice top | 07-apr-01 | 43º28'04.68'' | 008º18'47.17'' | rocky intertidal |
|  | MNCN 16.01/18413 | MNCN 16.01/16001 | 3D | Fornelos | crevice top | 07-apr-01 | 43º28'04.68'' | 008º18'47.17'' | rocky intertidal |
|  | MNCN 16.01/18414 | MNCN 16.01/16001 | 3A, B | Fornelos | crevice top | 07-apr-01 | 43º28'04.68'' | 008º18'47.17'' | rocky intertidal |
|  | MNCN 16.01/18415 | MNCN 16.01/16001 | 3C | Fornelos | crevice top | 07-apr-01 | 43º28'04.68'' | 008º18'47.17'' | rocky intertidal |
