# Supplementary material for: An integrative approach to the anatomy of Syllis gracilis Grube, 1840 (Annelida) using micro-computed X-ray tomography
Source: PeerJ. 2019 Jul 8;7:e7251. doi: 10.7717/peerj.7251 (PMC6622173; doi:10.7717/peerj.7251)
Supplement: Table S2 — Preparatory treatment of Syllis gracilis specimens examined with CLSM. [file peerj-07-7251-s002.docx]

| **TABLE 2** |  |  |  |  |  |  |
| --- | --- | --- | --- | --- | --- | --- |
| **Museum reference** | **Extracted from** | **Body region** | **Data observation** | **Triton X-100 (0.5%)** | **Phalloidin-FITC** | **time stain (min)** |
| MNCN 16.01/18412 | MNCN 16.01/16001 | entire body | 06-jun-18 | --- | 10µL/500µL | 90 |
|  |  | anterior end | 14-jun-18 | 5 min | 10µL/500µL | 90 |
| MNCN 16.01/18413 | MNCN 16.01/16001 | entire body | 06-jun-18 | --- | 5µL/500µL | 90 |
|  |  | entire body | 14-jun-18 | 5 min | 10µL/500µL | 90 |
| MNCN 16.01/18414 | MNCN 16.01/16001 | anterior end | 14-jun-18 | 10 min | 10µL/500µL | 120 |
| MNCN 16.01/18415 | MNCN 16.01/16001 | entire body | 14-jun-18 | 15 min | 5µL/500µL | 24 hours |
