# Supplementary material for: An integrative approach to the anatomy of Syllis gracilis Grube, 1840 (Annelida) using micro-computed X-ray tomography
Source: PeerJ. 2019 Jul 8;7:e7251. doi: 10.7717/peerj.7251 (PMC6622173; doi:10.7717/peerj.7251)
Supplement: Table S3 — Preparatory treatment of histological sectioned specimens of Syllis gracilis. [file peerj-07-7251-s003.docx]

| **TABLE 3** |  |  |  |  |  |
| --- | --- | --- | --- | --- | --- |
| **Museum reference** | **Extracted from** | **Number of slides** | **Plane of sectioning** | **Sectioning width** | **Stain** |
| MNCN 16.01/18342-18349 | MNCN 16.01/15999 | 8 | transversal | 8 µm | hematoxylin-eosin |
| MNCN 16.01/18350-18367 | MNCN 16.01/15999 | 18 | transversal | 8 µm | hematoxylin-eosin |
| MNCN 16.01/18368-18372 | MNCN 16.01/15999 | 5 | sagittal | 8 µm | hematoxylin-eosin |
| MNCN 16.01/18373-18382 | MNCN 16.01/15999 | 10 | transversal | 8 µm | hematoxylin-eosin |
| MNCN 16.01/18383-18398 | MNCN 16.01/16011 | 16 | transversal | 10 µm | Mallory |
| MNCN 16.01/18399-18404 | MNCN 16.01/16013 | 6 | transversal | 10 µm | Mallory |
