# Supplementary material for: An integrative approach to the anatomy of Syllis gracilis Grube, 1840 (Annelida) using micro-computed X-ray tomography
Source: PeerJ. 2019 Jul 8;7:e7251. doi: 10.7717/peerj.7251 (PMC6622173; doi:10.7717/peerj.7251)
Supplement: Table S4 — Preparatory treatment and scanning parameters of micro-CT studied specimens of Syllis gracilis. [file peerj-07-7251-s004.docx]

| **TABLE 4** |  |  |  |  |  |  |  |  |  |  |
| --- | --- | --- | --- | --- | --- | --- | --- | --- | --- | --- |
| **Museum reference** | **Extracted from** | **Scanned** | **Scanned length (mm)** | **Data scan** | **Stain** | **Dehydration** | **Voltage (kv)** | **Current (μA)** | **Filter** | **Pixel size (μm)** |
| MNCN 16.01/18405 | MNCN 16.01/16001 | w. a. | 9.53 | 26-jun-17 | no stain | HMDS | 55 kv | 165 μA | no filter | 3.73 |
|  |  |  |  | 07-jul-17 | iodine |  | 40 kv | 250 μA |  |  |
| MNCN 16.01/18406 | MNCN 16.01/16003 | w. a. | 1.74 | 14-dec-17 | no stain | HMDS | 55 kv | 165 μA | no filter | 0.70 |
| MNCN 16.01/18407 | MNCN 16.01/16007 | w. a. | 5.43 | 14-dec-17 | no stain | HMDS | 55 kv | 165 μA | no filter | 2.10 |
| MNCN 16.01/18408 | MNCN 16.01/16012 | w. a. | 1.85 | 12-dec-17 | no stain | HMDS | 55 kv | 165 μA | no filter | 0.81 |
| MNCN 16.01/18409 | MNCN 16.01/16000 | w. a. | 1.41 | 12-dec-17 | no stain | HMDS | 55 kv | 165 μA | no filter | 0.74 |
| MNCN 16.01/18410 | ---- | a. e. | 4.04 | 31-jan-17 | no stain | HMDS | 55 kv | 165 μA | no filter | 0.95 |
| MNCN 16.01/18411 | ---- | a. e. | 4.39 | 31-jan-17 | no stain | HMDS | 55 kv | 165 μA | no filter | 0.95 |
